# Unveiling the mechanism of action of a novel natural dual inhibitor of SARS-CoV-2 Mpro and PLpro with molecular dynamics simulations

**DOI:** 10.1007/s13659-024-00486-4

**Published:** 2025-01-04

**Authors:** Xiaoxia Gu, Xiaotian Zhang, Xueke Zhang, Xinyu Wang, Weiguang Sun, Yonghui Zhang, Zhengxi Hu

**Affiliations:** Hubei Key Laboratory of Natural Medicinal Chemistry and Resource Evaluation, School of Pharmacy, Tongji Medical College, Huazhong University of Science and Technology, Wuhan, 430030 People’s Republic of China

**Keywords:** SARS-CoV-2, PLpro, Mpro, Natural product, Dual inhibitor, Nano-channel, Antiviral agent

## Abstract

In the twenty-first century, we have witnessed multiple coronavirus pandemics. Despite declining SARS-CoV-2 cases, continued research remains vital. We report the discovery of sydowiol B, a natural product, as a dual inhibitor of SARS-CoV-2 main protease (Mpro) and papain-like protease (PLpro). Sydowiol B interacts with the nano-channel at the Mpro dimer interface and the PLpro active site. Molecular dynamics simulations suggest that sydowiol B inhibits Mpro by limiting active site expansion rather than inducing collapse. Furthermore, sydowiol B binding may amplify the fluctuation of two loops coordinating with the structural Zn^2+^ in PLpro, displacing Zn^2+^ from the zinc finger domain to the S2 helix. Sydowiol B and its analogue, violaceol I, exhibit broad-spectrum antiviral activity against homologous coronaviruses. Given the conservation of Mpro and PLpro, sydowiol B and violaceol I are promising leads for designing and developing anti-coronavirus therapies.

**Graphical Abstract:**

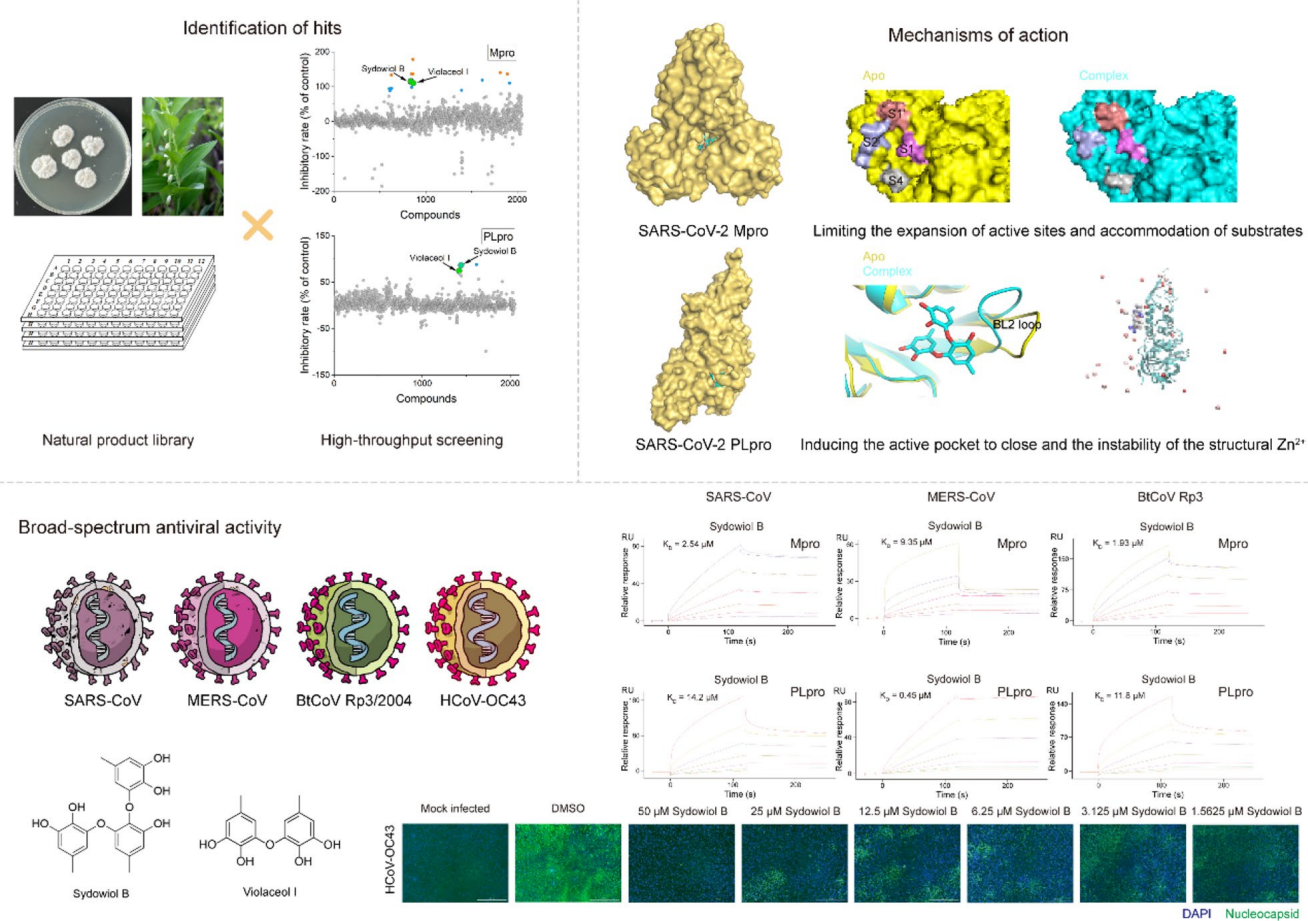

**Supplementary Information:**

The online version contains supplementary material available at 10.1007/s13659-024-00486-4.

## Introduction

The coronavirus disease 2019 (COVID-19) has been the most devastating pandemic of the twenty-first century, resulting in millions of deaths and widespread disruptions to society and economies. Excess mortality, a metric proposed by the World Health Organization (WHO) as a more accurate measure, is significantly higher than the reported COVID-19 mortality figures (e.g., 14.9 million vs. 5.5 million by the end of 2021), reflecting the far-reaching impacts of COVID-19 beyond its direct casualties. According to WHO reports, COVID-19 pandemic cost a staggering loss of 336.8 million years of life globally in just 2020 and 2021 [1]. This statistic underscores the profound and long-lasting consequences of the pandemic, extending beyond the immediate loss of life.

The causative pathogen, severe acute respiratory syndrome coronavirus 2 (SARS-CoV-2), is a single-stranded positive-sense RNA virus belonging to the *Betacoronavirus* genus, along with the related severe acute respiratory syndrome coronavirus (SARS-CoV) and middle east respiratory syndrome coronavirus (MERS-CoV). These three coronaviruses have caused independent pandemics in 2019, 2003, and 2012, respectively. Within the SARS-CoV-2 genome, two viral proteases, papain-like protease (PLpro) and main protease (Mpro), play indispensable roles in processing the viral polyprotein into 16 mature non-structural proteins (NSPs). These NSPs are essential for viral replication and propagation [2]. The two viral proteases, PLpro and Mpro, are highly conserved in coronaviruses and were proposed as therapeutic targets soon after the SARS-CoV-2 outbreak. Despite extensive efforts, only a few therapies, such as remdesivir and paxlovid (a combination of nirmatrelvir and ritonavir), are currently available. Moreover, due to the high mutation rates and genetic recombination events, vaccines (inactivated virus vaccines or nucleic acid vaccines) have rapidly lost their efficacy [3]. Considering the frequent emergence of *Betacoronaviruses* in recent years, more therapeutic options are urgently needed.

Natural products have been a crucial source for drug discovery. At the onset of the SARS-CoV-2 pandemic, researchers investigated antiviral agents from natural products [4–6], such as Shuanghuanglian preparations. Further crystallographic and cellular studies identified the active ingredient baicalein and its interaction with SARS-CoV-2 Mpro. Over the past several years, our group has been devoted to discovering structurally unique and bioactive natural products from various species, including *Aspergillus flavipes* [7], *Bipolaris* sp. TJ403-B1 [8], *Hypericum perforatum* [9], *Penicillium griseofulvum* [10], et al. We have built an in-house natural product entity library (approximately 19,000 compounds) and a virtual compound information library (approximately 47,000 compounds), which served as the foundation for this study.

Through high-throughput screening, we identified two natural products, sydowiol B and its analogue violaceol I, as dual inhibitors of SARS-CoV-2 Mpro and PLpro. Sydowiol B was found to bind to Mpro at the dimer interface, which is more conserved than the active site among SARS-CoV-2 variants. We verified the druggability potential of the nano-channel at the Mpro dimer interface. Moreover, sydowiol B and violaceol I exhibited broad-spectrum antiviral activity by targeting Mpro and PLpro from SARS-CoV, MERS-CoV, and the related Bat coronavirus Rp3/2004 (BtCoV Rp3/2004). Furthermore, against human coronavirus OC43 (HCoV-OC43), sydowiol B and violaceol I displayed more potent antiviral activity at micromolar concentrations, potentially due to their dual-targeting inhibition mechanism.

## Results

### Sydowiol B and violaceol I are dual-targeting antivirals for SARS-CoV-2

**Fig. 1:**
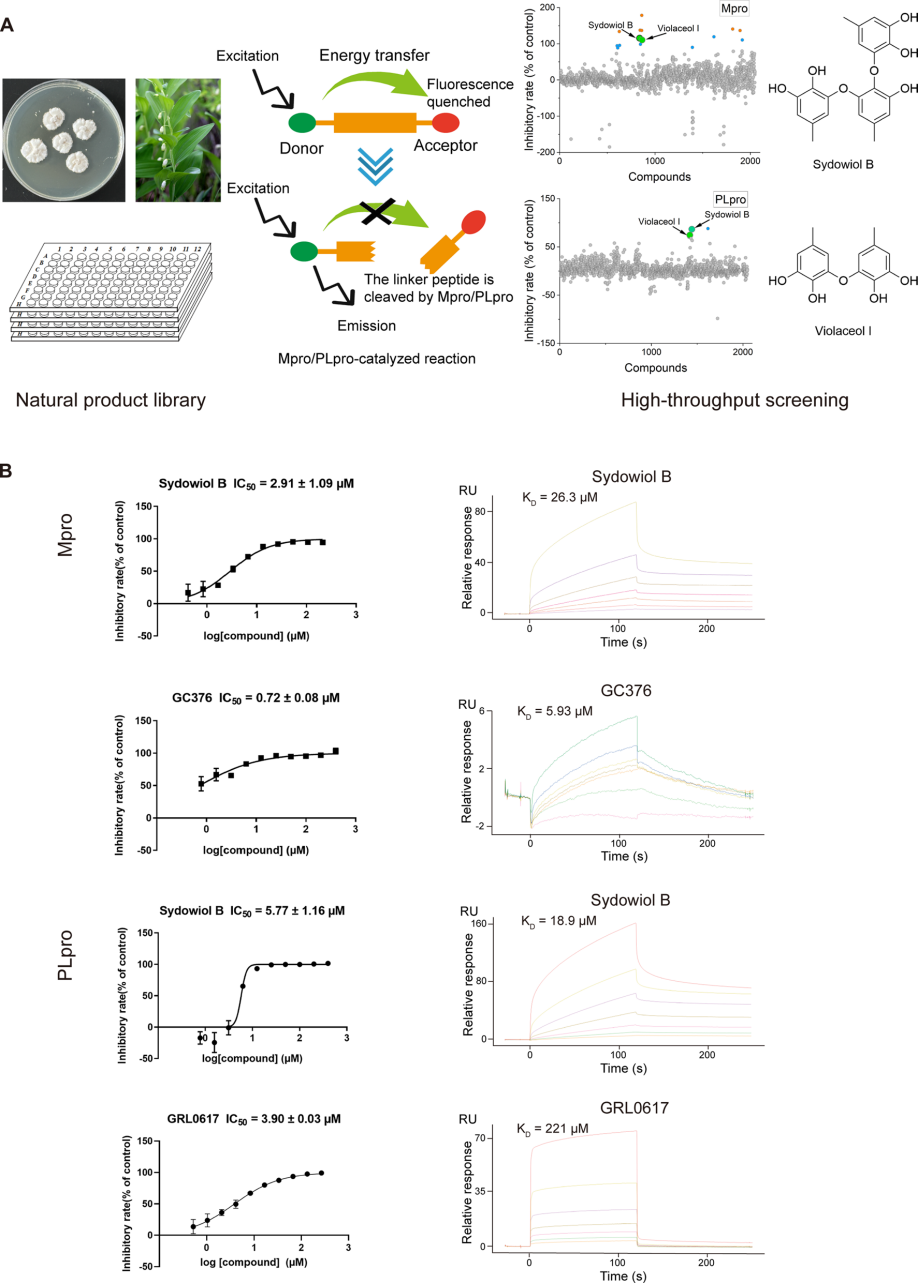
Sydowiol B and violaceol I are dual inhibitors of SARS-CoV-2 Mpro and PLpro. **A** Schematic representation of the high-throughput screening process that identified sydowiol B and violaceol I from an in-house natural product library (approximately 2000 compounds). The chemical structures of sydowiol B and violaceol I are shown. **B** Inhibitory activity (IC_50_ values) and binding affinity of sydowiol B against Mpro and PLpro. GC376 and GRL0617 were used as positive controls for Mpro and PLpro, respectively

Based on high-throughput screening, we identified two natural product hits, sydowiol B and its analogue violaceol I (Fig. 1A), from a subset of our in-house natural product library (approximately 2000 compounds). Both compounds exhibited dual inhibitory activity against the SARS-CoV-2 Mpro and PLpro. For Mpro and PLpro, the IC_50_ values of sydowiol B were 2.91 ± 1.09 μM and 5.77 ± 1.16 μM, respectively (Fig. 1B), while those of violaceol I were 8.27 ± 1.15 μM and 42.35 ± 1.02 μM, respectively (Supplementary Fig. S1). Notably, sydowiol B exhibited comparable activity to the reference compounds GC376 and GRL0617. We further confirmed the binding affinity between the two compounds and Mpro or PLpro. Sydowiol B and violaceol I both exhibited micromolar-scale interactions with the two proteases, comparable to GC376 and significantly better than GRL0617 (Fig. 1B, Supplementary Fig. S1).

### Molecular docking of sydowiol B with SARS-CoV-2 Mpro

**Fig. 2:**
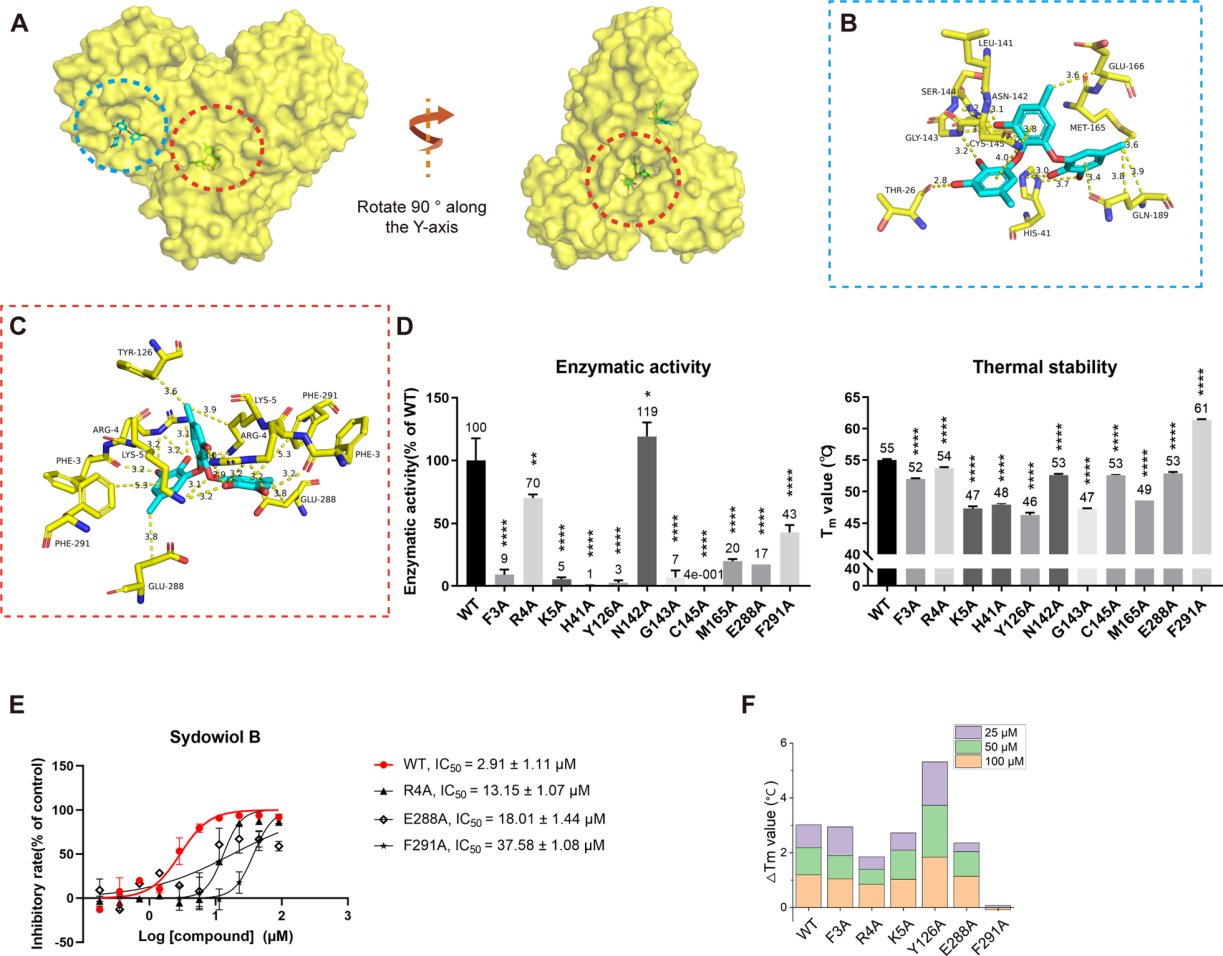
Molecular docking of sydowiol B with SARS-CoV-2 Mpro. **A** The surface of the structure 6WTM complexed with sydowiol B, highlighting two potential binding sites: the active site (blue circle) and the nano-channel (red circle). **B** The representative interaction and involved residues between sydowiol B and the active site of Mpro are illustrated using the PDB model 7LZT. The conformation of the ligand belongs to mono_group 2. **C** The representative interaction and involved residues between sydowiol B and the nano-channel of Mpro are illustrated using the PDB model 6WTM. The conformation of the ligand belongs to dimer_group 1. **D** The enzymatic activity and thermal stability (T_m_ value) of Mpro mutants (*P* values were calculated by comparing the mutants with WT). **E** The inhibition of sydowiol B against Mpro mutants derived from the nano-channel. **F** The effect of sydowiol B on the thermal stability (T_m_ value) of Mpro mutants derived from the nano-channel

To elucidate the possible binding site and interactions involved, we performed molecular docking of sydowiol B with SARS-CoV-2 Mpro. Previous research has revealed multiple conformations of SARS-CoV-2 Mpro and the equilibrium among these conformations, as well as possible intermediate states. Additionally, substrate or inhibitor binding can induce structural changes in the protein [11–14]. Considering these factors that could affect docking results, we selected multiple PDB structures as docking models, including 5R80, 5RE4, 7ALH, 7K0G, 7LMD, 7LZT, 6WTM, 6XHU, 7B3E, 7BB2, 7BE7, 7BGP, 7C2Q, and 7C2Y. These structures covered both the apo and complex states of Mpro, as well as different conformational changes induced by diverse inhibitors. According to the docking results, sydowiol B might bind to SARS-CoV-2 Mpro at two different sites: one in the active site where substrates and inhibitors typically bind, and the other at the nano-channel located between the two subunits of the Mpro dimer (Fig. 2A).

By comparing the conformations of the ligand, we further subgrouped the docking results within the two binding sites. For the active site, the docking conformations of sydowiol B could be sorted into three subgroups based on their similarity: mono_group 1, mono_group 2, and mono_group 3 (Fig. 2B, Supplementary Fig. S2A-C). Between mono_group 1 and mono_group 2, four residues were common: Asn142, Gly143, Cys145, and Met165. The third subgroup, mono_group 3, shared limited overlap with the other two subgroups, involving only the catalytic dyad (His41 and Cys145). Combining this information, we selected five residues for further mutation analysis: His41, Asn142, Gly143, Cys145, and Met165 (Fig. 2B).

Similarly, for the nano-channel binding site, the docking conformations of sydowiol B could be sorted into two subgroups: dimer_group 1 and dimer_group 2 (Fig. 2C, Supplementary Fig. S2D-E). By comparing the overlaps between dimer_group 1 and dimer_group 2, six residues were selected for further analysis: Phe3, Arg4, Lys5, Tyr126, Glu288, and Phe291 (Fig. 2C).

In total, 11 residues were selected for mutation analysis. As expected, most mutants lost considerable enzymatic activity (Fig. 2D), since the activity of Mpro is regulated by numerous residues in the substrate-binding site and extra domains [15–17]. Only five mutants (R4A, N142A, M165A, E288A, and F291A) retained measurable enzymatic activity, with N142A displaying a slight increase in enzymatic activity (approximately 20%) compared to the wild-type (WT) enzyme. Moreover, these mutants exhibited variable thermal stability. R4A and N142A had comparable activity and similar thermal stability to WT, while F291A exhibited significantly lower activity than WT but had a slightly increased melting temperature (T_m_) value. Despite their unmeasurable enzymatic activity, the remaining eight mutants displayed similar or lower thermal stability compared to WT. These results suggest that there is no clear correlation between thermal stability and enzymatic activity, at least in SARS-CoV-2 Mpro.

To verify the possible interaction between sydowiol B and Mpro, we measured the IC_50_ values of sydowiol B against Mpro mutants and T_m_ values of Mpro mutants with or without sydowiol B interference. Of the five mutants with measurable activity, N142A and M165A showed minor increases in the IC_50_ values of sydowiol B. In contrast, R4A, E288A, and F291A exhibited remarkably decreased inhibition from sydowiol B, with 4.52-fold, 6.19-fold, and 12.91-fold higher IC_50_ values compared to WT, respectively (Fig. 2E). In the thermal shift assay, four mutants from the nano-channel (R4A, K5A, E288A, and F291A) showed decreased ΔT_m_ values (Fig. 2E, Supplementary Table S1), especially F291A, indicating impaired interaction of sydowiol B with the corresponding mutants and suggesting that these residues possibly participated in the interaction between sydowiol B and Mpro WT. Additionally, there was a notable increase in the ΔT_m_ value of N142A, partly excluding the possibility of interaction between sydowiol B and Asn142. Combined with the results of the IC_50_ measurements, where sydowiol B exhibited nearly unchanged inhibition against mutants derived from the active site (N142A and M165A), we concluded that the binding site of sydowiol B with Mpro would be the nano-channel.

### Sydowiol B bound to SARS-CoV-2 Mpro at the nano-channel

**Fig. 3:**
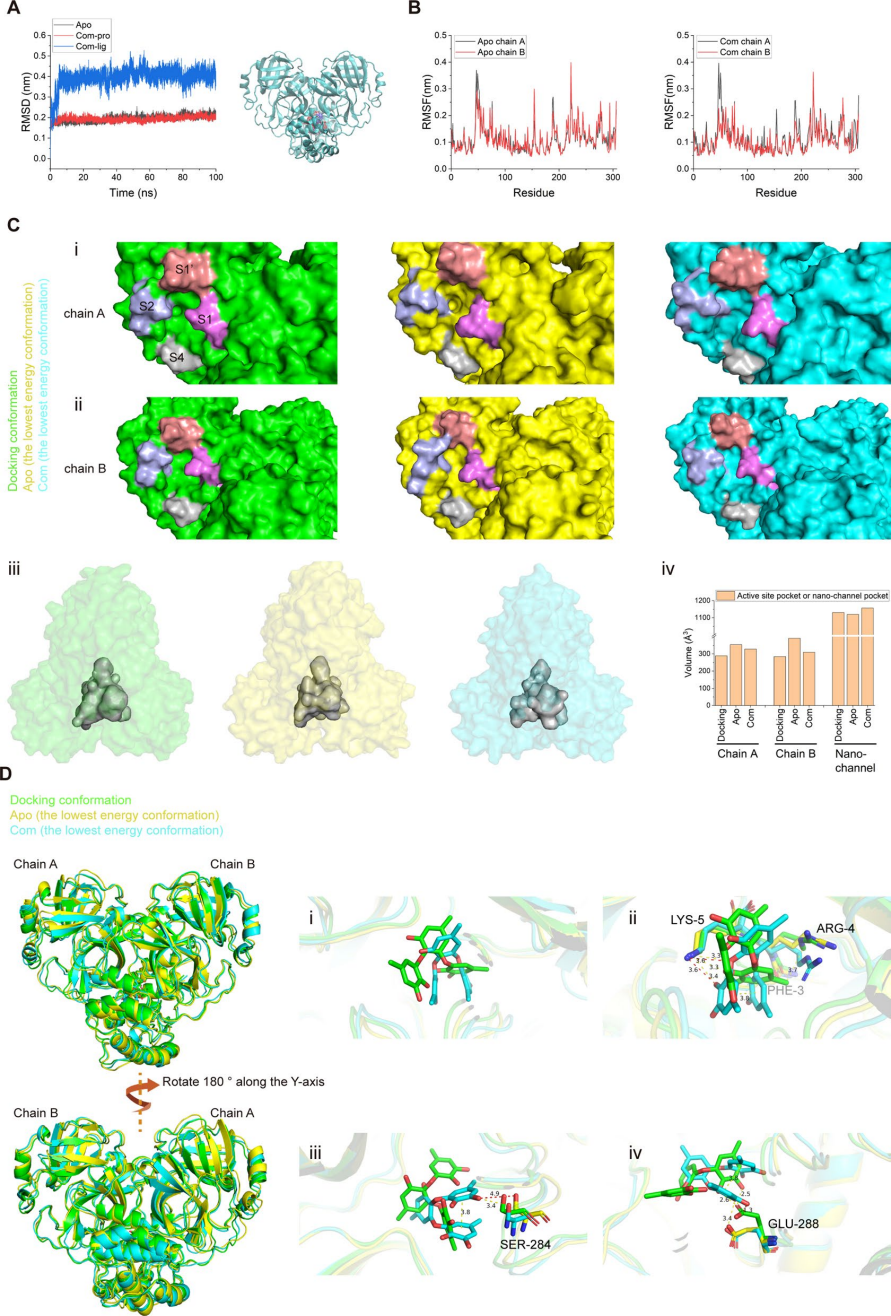
Sydowiol B bound to SARS-CoV-2 Mpro at the nano-channel. **A** The RMSD plot illustrates the stability of Mpro and the ligand sydowiol B in the apo (unbound) and com (complexed with sydowiol B) states, along with the trajectory of the ligand during the simulation after aligning the protein. **B** The RMSF plot represents the flexibility of residues in chain A and chain B of Mpro in the apo and com states. **C** This panel compares the size of the two active sites and the nano-channel in the docked conformation, apo state, and com state (the latter two represent the lowest energy conformations from principal component analysis): (i) the active site from chain A, (ii) the active site from chain B, (iii) the nano-channel, and (iv) a bar graph depicting the volume of these three sites in the respective states. **D** This panel illustrates the conformational changes induced by the binding of sydowiol B in the nano-channel, including the ligand itself and the surrounding residues

To inspect the binding situation of sydowiol B with SARS-CoV-2 Mpro in more detail, we conducted a 100 ns molecular dynamics (MD) simulation for both the apo and complex (com, Mpro complexed with sydowiol B) states, using the 6WTM model, which exhibited better structural integrity and quality as assessed by the PDB database. The corresponding docking result was used as the initial pose. The root-mean-square deviation (RMSD) plot revealed that after a short fluctuation (approximately 6 ns), the ligand sydowiol B was consistently held at the nano-channel, which could also be observed from the trajectory of the ligand after aligning Mpro (Fig. 3A). Additionally, we measured the distance between the ligand and three loops located in the nano-channel from different directions: residues 3–5 above the ligand, residues 284–286 beneath it, and residues 288–291 on the flank. The distances from all three directions showed that the ligand was held steady in the nano-channel (Supplementary Fig. S3A). Meanwhile, we discovered that the ligand progressively drifted away from chain A and approached chain B in all three directions. The radius of gyration (Rg) plot indicated that the structures of both apo and com remained compact during the MD simulation (Supplementary Fig. S3B). Further decomposition of Rg into three independent axes showed no significant difference in trends between apo and com (Supplementary Fig. S3C). The root-mean-square fluctuation (RMSF) plot and the distance between His41 and Cys145 revealed the asymmetry between the two chains of Mpro (Fig. 3B, Supplementary Fig. S3D), consistent with previous reports [18, 19]. It seemed that chain B adopted the "right conformation" necessary for catalysis, considering the distance between His41 and Cys145 [19], and sydowiol B significantly stabilized chain B with minimal influence on chain A (which showed only a momentary conformation transition after ligand binding but mostly remained in the unsuitable conformation). Particularly, residues around Asp48 (located at the S2 helix and forming the flank of the active site) and Tyr154 (located in a loop connecting the two β-sheets around the S1 and S4 loops) of chain B displayed significantly reduced fluctuation after sydowiol B binding.

We then calculated the size of the active site by measuring the distances between the S1' loop (residues 21–26) and the S4 loop (residues 167–170), as well as between the S1 loop (residues 139–144) and the S2 helix (residues 46–50). During the simulation process, the distance between S1' and S4 remained stable in both chains and both states. However, the distance between S1 and S2 in both chains of com displayed a remarkable shrinkage compared to apo (Supplementary Fig. S3E). It has been reported that the active site of Mpro is malleable, especially the S2 helix, and upon ligand binding, it gradually expands to accommodate the side chains of the ligands [20–22]. In contrast, the changes observed upon sydowiol B binding caused the active site to contract and restricted its plasticity for substrate binding, which might result in the limitation of catalytic activity.

Additionally, we measured the size of the nano-channel. Unsurprisingly, the nano-channel exhibited considerable expansion in three different measurements, including the distances between residues 288–291 (chain A) and residues 288–291 (chain B) (this distance was positively correlated with the width of the nano-channel), between residues 3–5 (chain A) and residues 284–286 (chain B), and between residues 3–5 (chain B) and residues 284–286 (chain A) (the latter two distances were positively correlated with the length and height of the nano-channel, respectively) (Supplementary Fig. S3F).

We further conducted principal component analysis (PCA) on both states. The sizes of the active site and the nano-channel were calculated in the resulting lowest energy conformations from PCA (Supplementary Fig. S3G) and the docking conformation. The volumes of the two active sites from the docking conformation seemed identical, as it was a crystal structure of Mpro in the apo state. The two active sites of apo, which were relatively free in solution compared to the tightly compact docking conformation, tended to expand in both chains, while the binding of sydowiol B inhibited this progress, particularly in chain B (Fig. 3C i, 3C ii, and 3C iv). The measurement of active site volume also supported the asymmetry of the two chains in Mpro, with chain B adopting the "right conformation" and showing more flexibility in the active site, which might be related to substrate accommodation. Further inspection of several structural markers of protomer activity, including the salt bridge between Glu166 and His72 and the π-π stacking between Phe140 and His163, supported that chain B was the active protomer. Chain A in apo retained one of the two interactions (Glu166-His172) and collapsed to a certain extent, while both interactions of chain A in com were disrupted after sydowiol B binding (however, Glu166 was not oriented towards His163 to form an interaction; it just rotated away from both His172 and His163) (Supplementary Fig. S3K). The two structural markers of the docking conformation remained and were nearly identical, along with the previously mentioned two identical active site volumes, which were similar to a previously reported co-crystal [23]. Notably, the active site of com still maintained the two interactions (Glu166-His172 and Phe140-His163), the same as apo. It seemed that sydowiol B did not induce the collapse of active sites but limited their expansion and accommodation of substrates. On the other hand, the size of the nano-channel in apo seemed almost uniform with the docking conformation, but that in com displayed slight expansion, which might be a result of sydowiol B binding (Fig. 3C iv).

Gmx_MMPBSA decomposition analysis was conducted for both states between the two chains and between Mpro and the ligand sydowiol B. Compared to the apo state, the binding energy between the two chains was reduced upon sydowiol B binding (ΔG_bind_ = 18 kcal/mol), particularly at Glu290 (chain A) and Arg4 (chain B), which formed a salt bridge crucial for maintaining the dimerization of Mpro. In contrast, the other pair of interactions between Glu290 (chain B) and Arg4 (chain A) seemed unaffected (Supplementary Fig. S3H, S3I). Sydowiol B formed a stable complex with Mpro, with a binding energy of -30.6 ± 0.09 kcal/mol (Supplementary Table S2), which was mainly contributed by residues from chain B, namely Phe3, Arg4, Lys5, Ser284, Glu288, and Phe291 (Supplementary Fig. S3J), consistent with experimental results. Further analysis of the apo and com conformations from PCA revealed the torsion of sydowiol B, which resulted in π-π stacking within the ligand (Fig. 3D i). Compared to the docking conformation, this torsion led to the loss of a hydrophobic interaction with Arg4 but an additional hydrogen bond with Lys5. The two hydrogen bonds with Lys5 in com were both inaccessible in apo (Fig. 3D ii). A similar situation was observed with Ser284, where the binding of the ligand pulled the residue inwards (Fig. 3D iii). The torsion of sydowiol B might cause a steric clash with Glu288, forcing it to rotate slightly more than in apo, resulting in the loss of one of the hydrogen bonds with Glu288 in the docking conformation (Fig. 3D iv).

To further understand the regulation of sydowiol B from the nano-channel to the active site, we used the webPSN (http://webpsn.hpc.unimore.it) webserver tool to investigate the structural communication within the sydowiol B-bound Mpro. As reported in the literature, there was an intense network present in Mpro (Supplementary Fig. S4A). When we filtered the network by the requirement "midway through the ligand", we found that sydowiol B regulated the active site of chain B perhaps through two directly interacted residues (Glu288 and Lys5) from chain B, which was consistent with the gmx_MMPBSA results, and a downstream pathway Glu290(B)-Arg4(A)-Tyr126(B)-Phe140(B)-His163(B) (Supplementary Fig. S4B). As for the active site of chain A, the allosteric modulation of sydowiol B was more complex, possibly through Arg4(B)-Lys137(A)-Arg131(A)-Asn133(A)-Ala194(A)-Phe185(A)-Phe181(A) and thereafter spreading. The directly interacted residue Arg4 (B) was still consistent with the gmx_MMPBSA results. Apart from the active site, sydowiol B also displayed regulation of residues from the dimer interface, such as Phe3(B), Arg4(A/B), Lys5(B), Ala7(A/B), Val125(B), Tyr126(B), Phe8(A), Lys137(A), and more, which might thus weaken the binding between the two chains (Supplementary Fig. S4A).

### Molecular docking of sydowiol B with SARS-CoV-2 PLpro

**Fig. 4:**
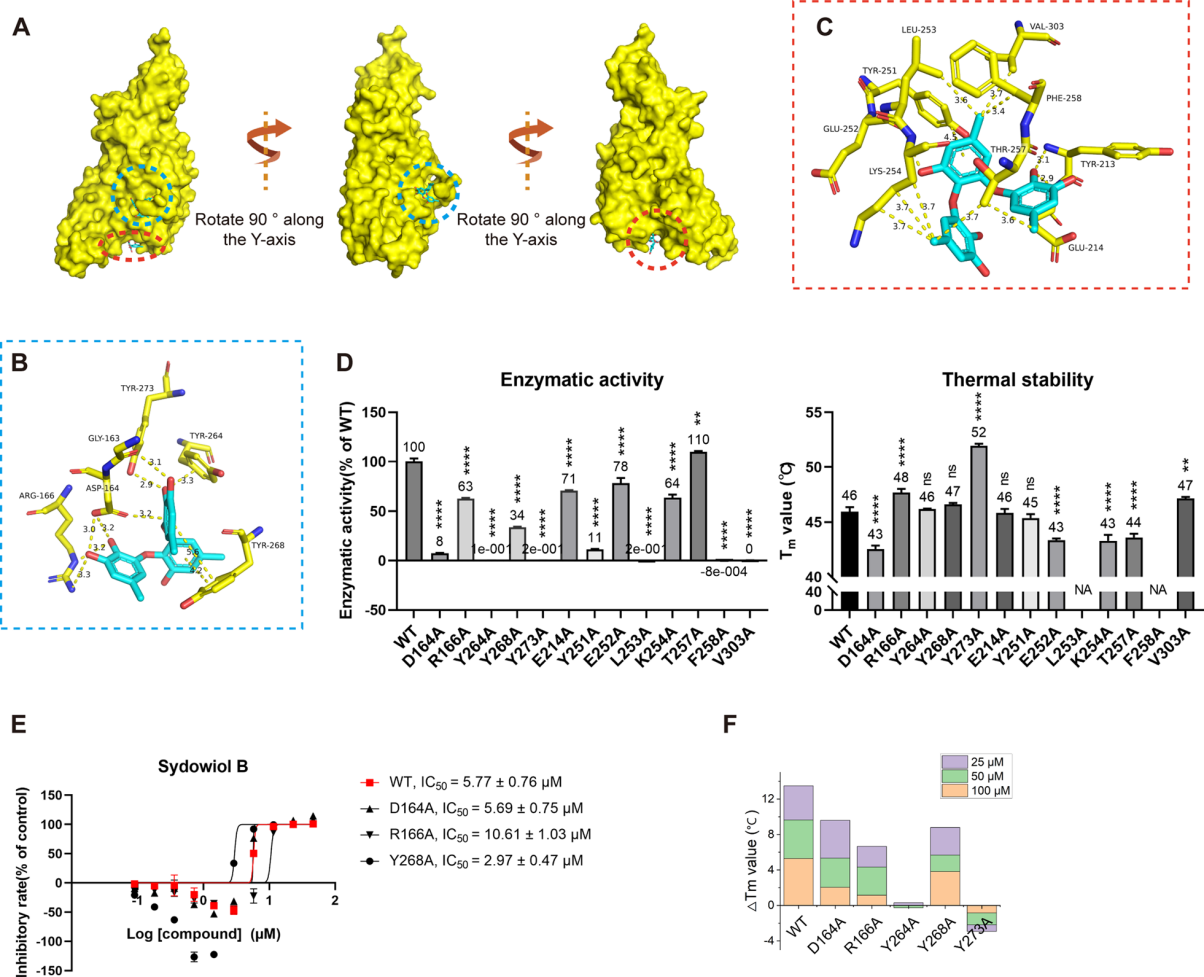
Docking results of sydowiol B with multiple PDB models of SARS-CoV-2 PLpro. **A** The surface representation of the model 8G62 complexed with sydowiol B highlights the two binding sites: the active site (blue circle) and the allosteric site (red circle). **B** The representative interactions and involved residues between sydowiol B and the active site of PLpro are illustrated using the PDB model 8G62. **C** The representative interactions and involved residues between sydowiol B and the allosteric site of PLpro are depicted using the PDB model 7JIW. **D** The enzymatic activity and thermal stability (melting temperature, T_m_) of PLpro mutants are shown (*P*-values were calculated by comparing with the wild-type enzyme). **E** The inhibitory effect of sydowiol B against PLpro mutants derived from the active site is presented. **F** The impact of sydowiol B on the thermal stability (T_m_) of PLpro mutants derived from the active site is illustrated

Similar to the approach used for Mpro, we performed multiple molecular docking simulations of sydowiol B with SARS-CoV-2 PLpro using various PDB structure models. In contrast to the results of Mpro, the predicted binding sites of sydowiol B on PLpro were diverse and scattered. Consequently, we selected the two most promising sites for further analysis: the active site and allosteric site (Fig. 4A).

Twelve out of the 25 docking results showed sydowiol B binding at the active site of PLpro, accounting for nearly half of the total results. Six residues (Gly163, Asp164, Arg166, Tyr264, Tyr268, and Tyr273) were consistently involved in the interaction across different docking models (Fig. 4B, Supplementary Fig. S5A). As Gly163 was predicted to interact with sydowiol B through its main chain carbonyl oxygen, it was excluded from further mutation analysis. The second binding site of sydowiol B on PLpro was located beneath the active site, resembling a U-shaped valley (Fig. 4A). Among the four docking results in this site, eight residues (Glu214, Tyr251, Glu252, Leu253, Lys254, Thr257, Phe258, and Val303) were consistently present and selected for further analysis (Fig. 4C, Supplementary Fig. S5B).

Among the 13 analyzed mutants, most exhibited impaired enzymatic activity, with Y264A, Y273A, L253A, F258A, and V303A displaying nearly unmeasurable activity levels. In contrast, the T257A mutant seemed unaffected, exhibiting a slightly increased activity (Fig. 4D). Regarding thermal stability, the majority of the mutants exhibited either unaffected or decreased stability compared to the wild-type enzyme. However, R166A, Y273A, and V303A showed a slight increase in T_m_ values, despite their compromised enzymatic activity. Notably, L253A and F258A exhibited a clear decrease in thermal stability, with their melting curves being unfittable (Fig. 4D).

The enzymatic activity assay results suggested that residues from both binding sites (i.e., Arg166 and Tyr251) might be involved in the interaction between sydowiol B and PLpro, as the corresponding mutants exhibited increased IC_50_ values (Fig. 4E, Supplementary Fig. S5C). However, these two mutants also displayed impaired enzymatic activity compared to the wild-type enzyme, which could partially contribute to the increased inhibition by the compound. To identify the authentic binding site, we employed a thermal shift assay. All five mutants derived from the substrate-binding site (D164A, R166A, Y264A, Y268A, and Y273A) showed decreased ΔT_m_ values, with Y264A and Y273A exhibiting the most significant reductions (Fig. 4F, Supplementary Table S3). These results collectively support the notion that sydowiol B interacts with PLpro at the active site. Some mutants from the U-shaped binding site also exhibited slightly decreased ΔT_m_ values (Supplementary Fig. S5D, Table S3), which might be attributed to overall structural effects induced by sydowiol B.

### Sydowiol B bound to SARS-CoV-2 PLpro at the active site

**Fig. 5:**
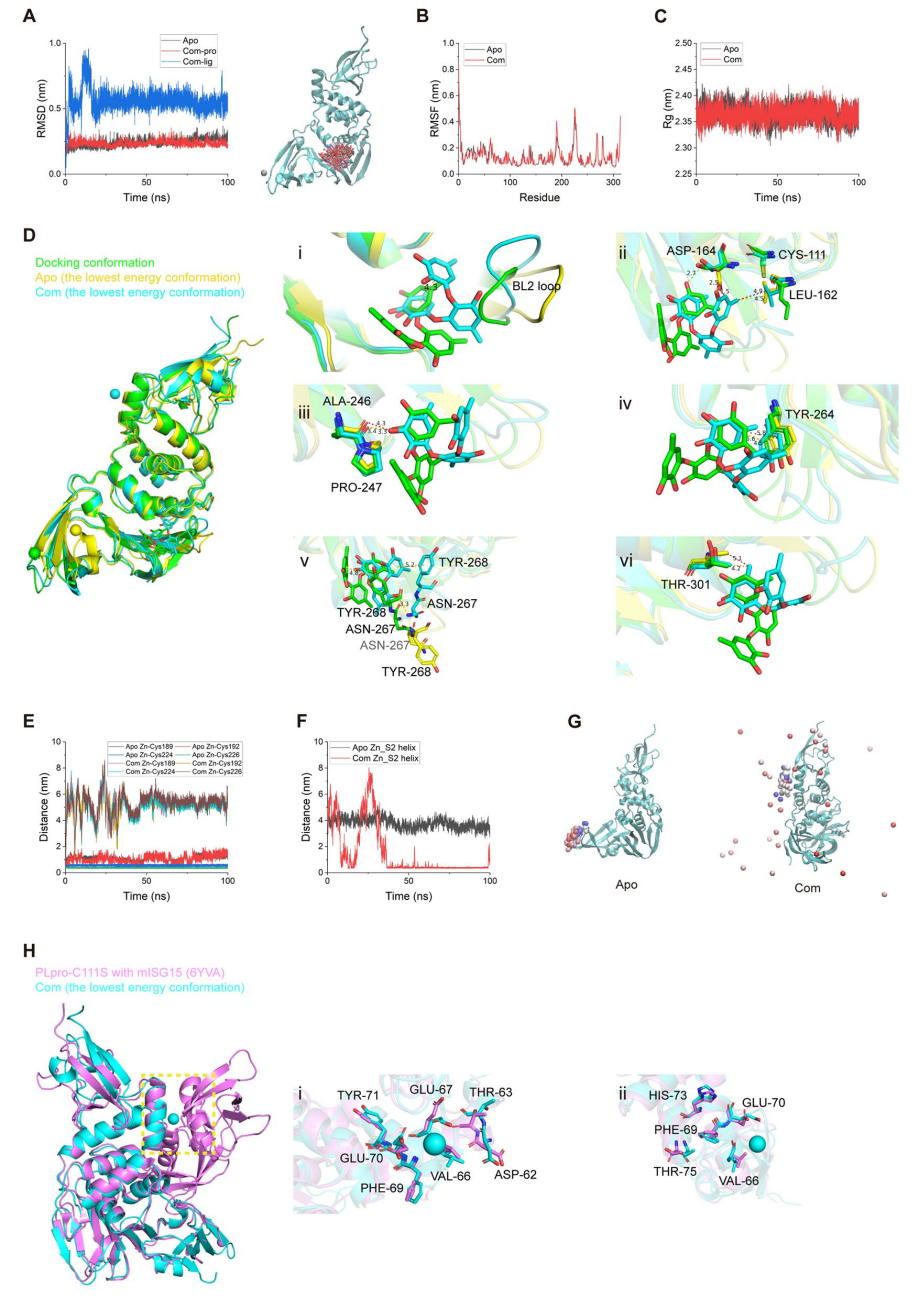
Sydowiol B bound to SARS-CoV-2 PLpro at the active site. **A** The RMSD plot depicts the stability of PLpro and the ligand sydowiol B in the apo (unbound) and com (complex with sydowiol B) states, along with the trajectory of the ligand during the simulation after aligning the protein. **B** The RMSF plot represents the flexibility of residues of PLpro in the apo and com states. **C** The Rg plot illustrates the compactness of PLpro in the apo and com states. **D** This panel shows the conformational changes induced by the binding of sydowiol B in the active site, including the ligand itself and the surrounding residues. **E** The distances between the Zn^2+^ ion and its four coordinating cysteine residues (Cys189, Cys192, Cys224, and Cys226) are presented. **F** The distance between the Zn^2+^ ion and the S2 helix in the apo and com states is depicted. **G** The trajectory of the Zn^2+^ ion during the simulation after aligning PLpro in the apo and com states is shown. **H** This panel illustrates the conformational changes of residues from the S2 helix induced by the binding of sydowiol B

To further confirm the interaction between sydowiol B and PLpro, we conducted a 100 ns MD simulation in the apo (unbound) and com (complexed with sydowiol B) forms, respectively. The model 8G62 was selected due to its better structural integrity and quality, as assessed by the Protein Data Bank (PDB). The corresponding docking result was used as the initial pose. The RMSD plot revealed that the protein reached equilibrium quickly (around 2 ns) in both apo and com forms. Similarly, after approximately 25 ns of fluctuation, the ligand sydowiol B also reached a steady state (Fig. 5A). The distance between sydowiol B and Tyr264 (approximately 2.5 Å) was well-sustained (Supplementary Fig. S6A), and the ligand trajectory showed that it remained inside the active site pocket throughout the entire simulation (Fig. 5A). Beginning from 25 ns, we calculated the RMSF of residues, which revealed that apart from the terminal residues, four loops displayed particularly pronounced fluctuations: two loops coordinating the structural Zn^2+^ (one loop centered on Cys189/Cys192, and the other centered on Cys224/Cys226), the BL2 loop (centered on Tyr268), and the loop centered on Glu280 (Fig. 5B). Furthermore, the former two loops exhibited more fluctuation when sydowiol B bound, while the other two loops seemed unaffected. The Rg plot of apo and com displayed steady and similar movements, confirming that the protein fold was well-sustained (Fig. 5C). However, when we decomposed the total Rg into three independent axes, we found that although the fluctuation level was similar between apo and com, apo displayed a more compact stacking in the X-axis but a looser stacking in the Z-axis compared to com (Supplementary Fig. S6B), which might result from the accommodation of the ligand in the X-axis and the inward contraction of the BL2 loop in the Z-axis in the com form. We further compared the size of the active pocket by measuring the distance between the BL2 loop (represented by Tyr268 and Gln269) and three residues (Leu162, Gly163, and Asp164) that formed a narrow tunnel leading to the active Cys111. In the apo form, the pocket preferred to adopt an open conformation, while in the com form, after a brief period of expansion (approximately 25 ns), it was well-sustained in a closed conformation (Supplementary Fig. S6C). However, this period of expansion did not result in pulling the ligand into the pocket (as can be seen from the distance between the ligand and residues 162–164, Supplementary Fig. S6A) but mainly arose from the conformational change of the ligand.

We conducted principal component analysis (PCA) and gmx_MMPBSA decomposition analysis to further inspect the detailed conformational changes induced by sydowiol B (Supplementary Fig. S6D). The gmx_MMPBSA analysis revealed that the binding of sydowiol B to PLpro was stable (ΔGibbs = -25.29 ± 0.07 kcal/mol, Supplementary Table S4), and the primary residues participating in the interaction included Leu162, Asp164, Ala246, Pro247, Tyr264, Asn267, Tyr268, and Thr301 (Supplementary Fig. S6E), which corresponded to the results of the thermal shift assay. The lowest energy conformation resulting from PCA was extracted and compared between apo and com, as well as the docking conformation. The PDB model (8G62) in the docking conformation was a co-crystal structure of PLpro with inhibitors, where the inhibitors adopted a relatively planar configuration. Compared to the docking pose, sydowiol B in com rotated approximately 90 degrees around the Y-axis, forming a π-π stacking between two benzene rings. The BL2 loop preferred a closed position in both the docking conformation and com. However, affected by the rotation of sydowiol B, the BL2 loop in com was forced to move away to avoid steric clash with the ligand, whereas in apo, it adopted an expanded conformation (Fig. 5D i). Compared to the docking conformation, the carbonyl of Asp164 flipped to sustain the hydrogen bond with the phenolic hydroxyl group of sydowiol B but from a different benzene ring, while the carboxyl group of the apo might induce a spatial clash with sydowiol B (Fig. 5D ii), reflecting the induced accommodation of the ligand. Leu162 of apo and com both flipped towards Cys111, consistent with previous reports about its role in blocking access to the catalytic Cys111. However, influenced by sydowiol B, the hydrophobic side chain of Leu162 moved slightly towards the methyl group of the ligand (Fig. 5D ii), similar to the main-chain carbonyl of Ala246, which formed a hydrogen bond with sydowiol B (Fig. 5D iii), and the side-chain benzene ring of Tyr264, which formed a π-π stacking with sydowiol B (Fig. 5D iv). Affected by the 90-degree rotation of sydowiol B, the side chain of Asn267 in com flipped towards the ligand to form an additional hydrogen bond, whereas in apo, it continued moving away from the docking conformation. The π-π stacking between sydowiol B and the side chain of Tyr268 was well-sustained in com (Fig. 5D v). The side chain of Thr301 between the docking conformation and com was practically unchanged, but considering the rotation of sydowiol B, it formed an additional hydrophobic interaction with the ligand, while in apo, it further moved away (Fig. 5D vi).

On the other hand, we found that the structural Zn^2+^ in com became unstable and could not settle in the zinc finger domain. Correspondingly, the Zn^2+^ in apo never moved outside the zinc finger, although the coordinate bonds between Zn^2+^ and Cys189/Cys192 were disrupted, and only the connection between Zn^2+^ and Cys224/Cys226 was sustained (Fig. 5E, 5G). In com, after approximately 36 ns of fluctuation, Zn^2+^ settled well in the S2 helix (Fig. 5F, 5G). After calculating the vacuum electrostatics of PLpro (in both apo and com forms), we found that the S2 helix displayed a significantly negatively charged surface (Supplementary Fig. S6H), which might be the reason for Zn^2+^ settling there. We further measured the distance between Zn^2+^ and individual residues of the S2 helix, and the negatively charged residues (Asp62, Glu67, and Glu70) and their surroundings exhibited more contacts and shorter distances than others (Supplementary Fig. S6G). Considering its role in binding ISG15 and di-ubiquitin, we further investigated the conformational changes in the S2 helix induced by sydowiol B and the resultant unstable Zn^2+^. First, the negatively charged residues (Asp62, Glu67, and Glu70) displayed visible rotation or flip towards Zn^2+^, accompanied by their adjacent residues (Fig. 5H i). It has been reported that some residues (Val66, Phe69, Glu70, His73, and Thr75) play an important role in mediating the interaction of PLpro with either ubiquitin or ISG15 [24, 25], and we inspected the influence of sydowiol B on these residues. There were also clear shifts in the positions of these residues compared to the ISG15-bound PLpro (Fig. 5H ii). Considering that sydowiol B already occupied the substrate-binding pocket, the resultant migration of Zn^2+^ and conformational changes of residues in the S2 helix might further create an unfavorable environment for ISG15 or ubiquitin binding.

To confirm that the migration of Zn^2+^ was induced by sydowiol B binding, we conducted a 100 ns MD simulation of PLpro complexed with GRL0617 (com2). It was revealed that similar to sydowiol B, GRL0617 also induced more fluctuation of the two loops around Cys189/Cys192 and Cys224/Cys226 compared to apo (Supplementary Fig. S7B). Differently, the structural Zn^2+^ was well settled in the zinc finger domain, similar to apo (Supplementary Fig. S7G, S7H), which proved the specific influence of sydowiol B on Zn^2+^. Another notable point was that GRL0617 was not well held in the pocket (Supplementary Fig. S7A). Even though the overall fold was well sustained during the simulation, the protein in com2 exhibited more fluctuation in three independent coordinate axes than apo or the sydowiol B-bound com (Supplementary Fig. S7C, S7D). The active pocket was expanded similarly to that in apo instead of being closed as in the sydowiol B-bound com (Supplementary Fig. S7E).

It has been reported that the zinc finger domain is important for structural stability and proteolytic activity [26]. Apart from competitively occupying the active site, the allosteric effect of sydowiol B on the zinc finger and subsequent S2 helix might further intensify inhibition against PLpro. We also investigated the structural communication inside PLpro after sydowiol B binding using the webPSN server. It can be seen that the global network inside PLpro was less intense, but the network induced by sydowiol B was more interlaced compared to Mpro (Supplementary Fig. S8A, S8B). The allosteric effect was mediated through residues in the palm domain and reached the loop around Cys224/Cys226 (namely Gln237) and the β-sheet around the Cys189/Cys192 loop (namely Arg183). The ligand also showed regulation in residues from the thumb domain and UBL domain, such as Leu64, Thr72, Val11, and His17.

### The broad-spectrum antiviral activity of sydowiol B against homologous coronaviruses

To explore the broad-spectrum antiviral activity of sydowiol B, we tested its inhibition on Mpro and PLpro from three homologous coronaviruses: SARS-CoV, MERS-CoV, and BtCoV Rp3/2004 (also known as SARS-like coronavirus Rp3).

**Fig. 6:**
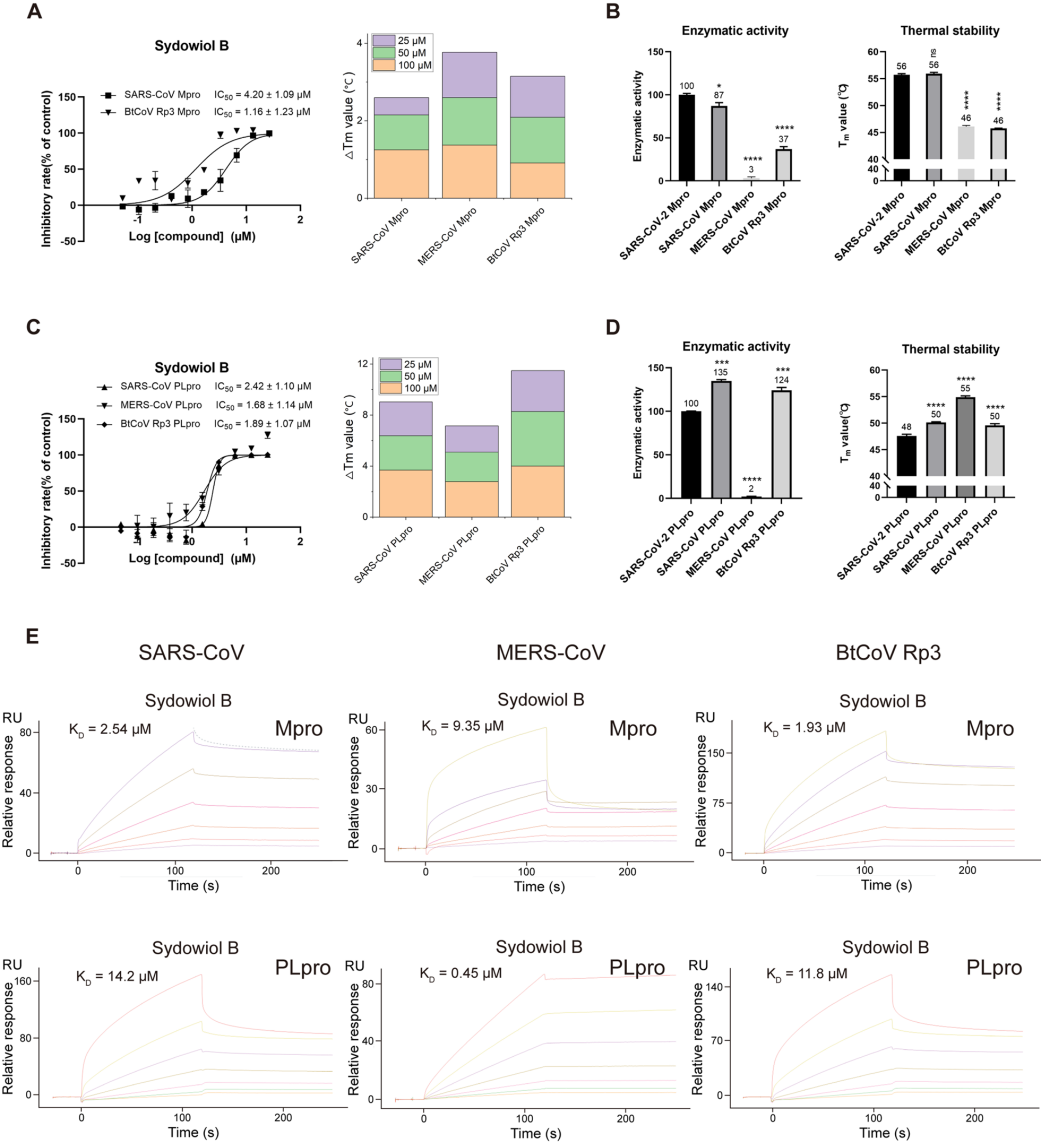
The broad-spectrum antiviral activity of sydowiol B. **A** The effect of sydowiol B on the enzymatic activity and thermal stability of Mpro from homologous coronaviruses is depicted. **B** The enzymatic activity and thermal stability of Mpro from homologous coronaviruses are shown (*P*-values were calculated by comparing with SARS-CoV-2 Mpro). **C** The effect of sydowiol B on the enzymatic activity and thermal stability of PLpro from homologous coronaviruses is illustrated. **D** The enzymatic activity and thermal stability of PLpro from homologous coronaviruses are presented (*P*-values were calculated by comparing with SARS-CoV-2 PLpro). **E** The binding affinity of sydowiol B with Mpro and PLpro from homologous coronaviruses is shown

Compared to SARS-CoV-2 Mpro, sydowiol B showed similar inhibition on SARS-CoV Mpro and even better outcomes against BtCoV Rp3 Mpro in the enzymatic activity assay (Fig. 6A). Its inhibition on MERS-CoV Mpro was not verified since the activity was unmeasurable under experimental conditions (Fig. 6B). Additionally, sydowiol B showed a similar effect on the thermal stability of MERS-CoV Mpro and BtCoV Rp3 Mpro, but to a slightly lesser extent on SARS-CoV Mpro. Meanwhile, we found that SARS-CoV Mpro exhibited comparable enzymatic activity to SARS-CoV-2 Mpro, but BtCoV Rp3 Mpro harbored only 37% activity of SARS-CoV-2 Mpro (Fig. 6B), which has just one mutation at residue 170 (G170E) compared to SARS-CoV Mpro. Moreover, the thermal stability of BtCoV Rp3 Mpro also presented a notable decrease, from 55.92 °C of SARS-CoV Mpro to 45.75 °C of BtCoV Rp3 Mpro. The less homologous MERS-CoV Mpro displayed clear decreases in both enzymatic activity and thermal stability (Fig. 6B, Supplementary Table S5). In the surface plasmon resonance assay, sydowiol B displayed a micromolar-level affinity with the three homologous Mpro, comparable to GC376, with K_D_ values of 2.54 μM for SARS-CoV, 9.35 μM for MERS-CoV, and 1.93 μM for BtCoV Rp3, respectively (Fig. 6E, Supplementary Table S6).

Regarding PLpro, sydowiol B exhibited more potent inhibition on PLpro from the three homologous coronaviruses compared to SARS-CoV-2 (Fig. 6C). Additionally, MERS-CoV PLpro showed clearly impaired activity compared to other coronaviruses, similar to Mpro, but with slightly increased thermal stability (Fig. 6D). Both SARS-CoV PLpro and BtCoV Rp3 PLpro showed increased activity and thermal stability compared to SARS-CoV-2 PLpro. Moreover, sydowiol B showed a similar influence on the thermal stability of SARS-CoV-2 PLpro and BtCoV Rp3 PLpro, but to a lesser extent on SARS-CoV PLpro and MERS-CoV PLpro (Fig. 6C, Supplementary Table S7). In the surface plasmon resonance assay, sydowiol B displayed a more potent affinity than GRL0617 against PLpro from the three coronaviruses. K_D_ values were 14.2 μM for SARS-CoV PLpro, 0.45 μM for MERS-CoV PLpro, and 11.8 μM for BtCoV Rp3 PLpro, respectively (Fig. 6E, Supplementary Table S8).

Violaceol I also exhibited broad-spectrum antiviral activity against Mpro and PLpro from the three homologous coronaviruses, with comparable inhibition to sydowiol B and even more potent affinity (Supplementary Fig. S9, Tables S6 and S8).

### The antiviral activity of sydowiol B and violaceol I in vitro

**Fig. 7:**
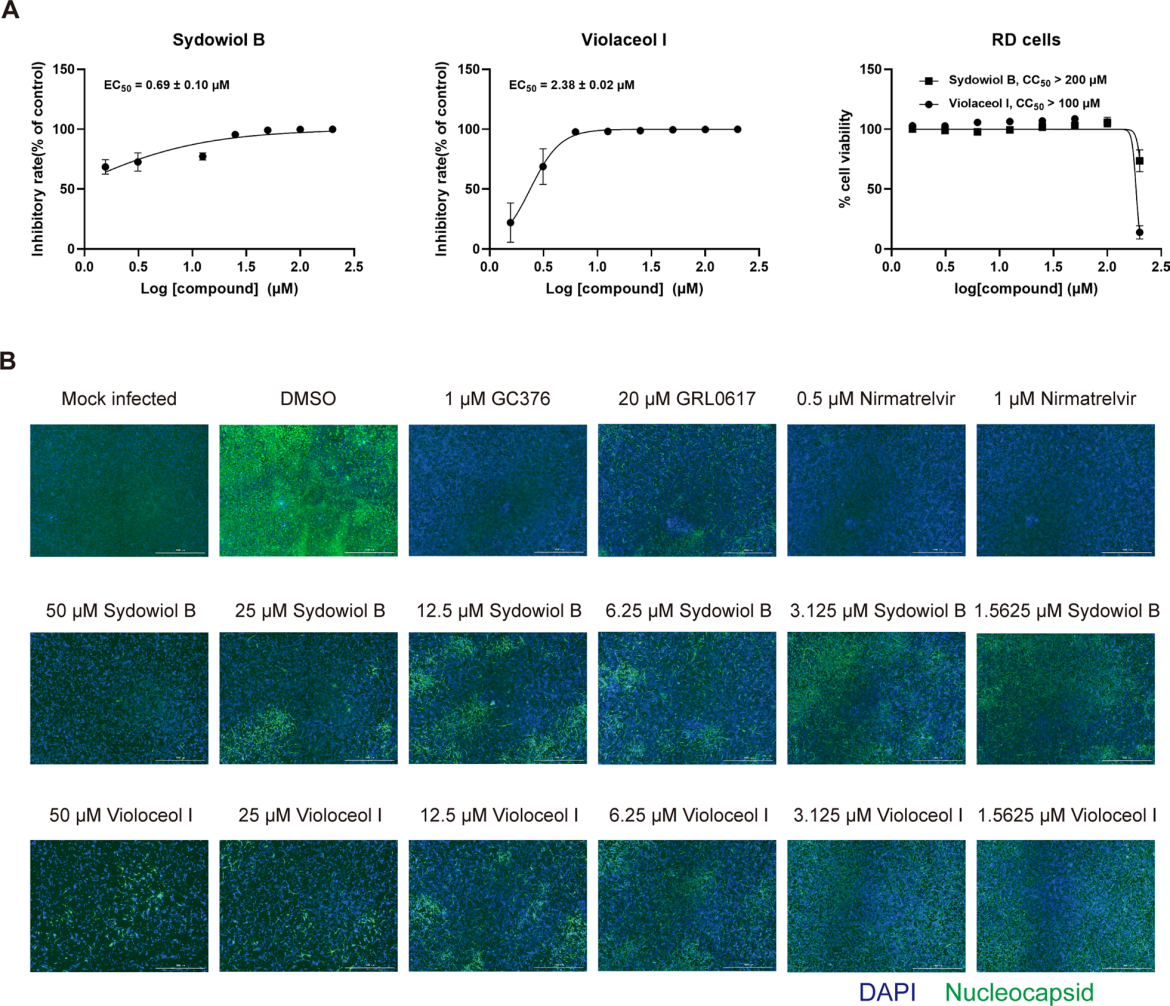
The in vitro antiviral activity of sydowiol B and violaceol I. **A** The inhibitory effect of sydowiol B and violaceol I against the human coronavirus OC43 (HCoV-OC43) is demonstrated through quantitative reverse transcription-polymerase chain reaction (qRT-PCR) analysis. **B** The inhibition of HCoV-OC43 by these compounds is confirmed by immunofluorescence assay (IFA)

To further verify the antiviral activity of sydowiol B and violaceol I, we tested their inhibition on a homologous coronavirus, HCoV-OC43, in vitro. qRT-PCR analysis revealed that both compounds displayed potent inhibition against HCoV-OC43, with EC_50_ values of 0.69 ± 0.10 µM for sydowiol B and 2.38 ± 0.02 µM for violaceol I (Fig. 7A). Moreover, the two compounds significantly decreased the expression of HCoV-OC43 nucleocapsid (N) protein, as revealed by immunofluorescence assay (IFA) (Fig. 7B). Sydowiol B and violaceol I showed no influence on cell viability at concentrations up to 100 µM (Fig. 7A, Supplementary Table S9).

## Discussion

From 2019 to 2022, SARS-CoV-2 caused a massive economic and public health burden. Although it seems to be receding from daily life, its far-reaching impact persists, and the trauma it caused remains to be dealt with, not just in terms of mortality but in all aspects it affected directly or indirectly. As we focus on economic reconstruction, persistent and thorough investigation into SARS-CoV-2 remains necessary, as the experience gained from SARS-CoV and MERS-CoV has already provided significant assistance in the SARS-CoV-2 pandemic.

In this study, we found two natural products, sydowiol B and its analogue violaceol I, to be dual inhibitors of SARS-CoV-2 Mpro and PLpro, with IC_50_ values of 2.91 µM and 8.27 µM for Mpro, and 5.77 µM and 42.35 µM for PLpro, respectively. To further reveal the mechanism of action of sydowiol B against SARS-CoV-2 Mpro and PLpro, we conducted molecular docking for each target. However, selecting an appropriate docking model is a crucial step. Proteins and other macromolecules are dynamic and exist in equilibrium among diverse states, conducting their biological functions [27, 28]. For Mpro, there is an equilibrium between monomer and dimer, as well as variable intermediate states [12, 14]. However, the solved structure is usually a static snapshot, an average state against a cluster of molecules from the sample. Additionally, an induced-fit effect occurs when compounds bind [11]. Another aspect potentially affecting protein conformations is the condition of cultivation, purification, and the construction strategy of recombinant plasmids, which could leave different residues at the terminus [29, 30]. Consequently, some researchers employed an ensemble docking approach, where models consisted of different states, such as apo-Mpro, inhibitor-bound Mpro, and conformations from MD simulations [31–33]. Similarly, we conducted multiple molecular dockings of sydowiol B against SARS-CoV-2 Mpro and PLpro, including apo and various inhibitor-bound states from different PDB models, and further grouped docking results by the presumed binding sites. The overlapped residues at each site were selected for further verification.

Based on results from mutation analysis, we conducted 100 ns MD simulations of Mpro and PLpro in apo and compound-bound states, respectively. It was revealed that sydowiol B stably bound to the Mpro nano-channel and exerted allosteric regulation on the active site, particularly that of the active chain B. Moreover, sydowiol B did not induce the collapse of the active site but constrained its expansion for substrate accommodation. It also regulated residues from the dimer interface, thus weakening the binding energy between the two chains. As for PLpro, sydowiol B bound to the active site and induced the BL2 loop to close instead of opening in the apo state. Furthermore, its binding enhanced the fluctuation of two loops around the structural Zn^2+^ and disrupted the connection between Zn^2+^ and coordinating cysteines. This interference on Zn^2+^ was specifically induced by sydowiol B, which was not observed in the GRL0617-bound PLpro. Influenced by electrostatic interactions, Zn^2+^ subsequently settled beside the S2 helix, which might further interrupt the binding of ISG15 or di-ubiquitin.

In the mutation analysis of SARS-CoV-2 Mpro, we found that the N142A mutant exhibited slightly improved activity, consistent with previous reports [34, 35]. We also revealed that the thermal stability of N142A was comparable to WT. Previous research identified resistance of N142A or N142S variants against nirmatrelvir, GC376, and ensitrelvir [34, 35], which we presumed might be partly due to the improved enzymatic activity and unaffected stability of these Mpro mutants since no loss of interaction was observed. Other mutants with impaired activity demonstrated distinct thermal stability, supporting the conclusion that there is no direct relationship between thermal stability and catalytic activity [36]. Similarly, in the mutation assay of SARS-CoV-2 PLpro, the Y273A mutant displayed significantly increased thermal stability, followed by the R166A and V303A mutants, all of which showed compromised catalytic activity. In contrast, the T257A PLpro mutant exhibited slightly increased activity compared to WT, with only minimally decreased thermal stability. Considering the lack of reports on this mutant, we investigated whether SARS-CoV-2 variants harbored this mutation. Using the CoVariants website (https://covariants.org/), we found a related variant 20 J (Gamma, V3), also known as Lineage P.1, harboring a T1820I mutation in ORF1a (i.e., T257I in PLpro), which was first detected in Brazil and earliest sampled on 22 September 2022, later spreading to 113 countries. Moreover, three variants of primary concern harboring mutations located around Thr257 of PLpro were identified: 21 K (Omicron) (Lineage BA.1) with T259I, 22F (Omicron) (Lineage XBB) with G256S, and 23A (Omicron) (Lineage XBB.1.5) with G256S. The three residues (Thr257, Thr259, and Gly256) are all located at a loop in the palm domain of PLpro. How these mutations affect the conformation and catalytic activity of PLpro and whether mutations occurring in this loop facilitate the spread of SARS-CoV-2 remain to be investigated.

Shortly after the structure determination of SARS-CoV-2 Mpro, it was proposed that several allosteric sites would be discretely presented in Mpro, as revealed by structural biology or in silico studies [37–42]. Due to the extensive interaction between the two subunits, Mpro harbors a relatively large dimer interface, and its activity is broadly regulated. The dimer interface is more conserved than the active site or other allosteric sites on the surface, thus compounds targeting it would be more resistant to Mpro variants [43]. Among these diverse allosteric sites, the nano-channel was proposed from SARS-CoV Mpro in 2005 [17] and also exists in SARS-CoV-2 Mpro, albeit more compact [44]. Recent development of SARS-CoV-2 Mpro inhibitors also disclosed considerable hits binding at this site, mainly identified through virtual screening or other computational algorithms or tools [37–39, 41]. Here, we experimentally proved that compounds could inhibit SARS-CoV-2 Mpro by binding at the nano-channel, confirming the accessibility and druggability of this allosteric site. We further checked the conservation of residues surrounding the nano-channel (the N-terminal five residues SGFRK, Asn214, and the region Glu288-Asp289-Glu290-Phe291 jointly form its flank, Ser284-Ala285-Leu286 constitute the bottom, and Arg298-Gln299 from the C-terminus reside around, while the top of the nano-channel is open and connected with the inner cavities of Mpro). Among 27 sequences of Mpro from the UniProt database (covering diverse coronaviruses), the residues in the flank of the nano-channel remain highly conserved, while those from the bottom display some variability. The C-terminal region Arg298-Gln299 exhibits considerable diversity (Supplementary Fig. S10A). However, when we retrospect this region to the structure of Mpro, it seems that the highly variable residue Arg298 does not directly form either side of the nano-channel (Supplementary Fig.S10B). Gln299 is highly conserved, but its role in the dimerization of Mpro possibly comes from the stabilization of the N-terminus and it still has no direct contact with the nano-channel (Supplementary Fig.S10C). Thus, the broad-spectrum antiviral activity of sydowiol B against Mpro from different coronaviruses, i.e., SARS-CoV-2, SARS-CoV, MERS-CoV, and BtCoV rp3, and possibly many more yet to be verified, is reasonable.

Apart from Mpro, sydowiol B targeted the active site of SARS-CoV-2 PLpro, and the dual-targeting inhibition might be the reason for considerably improved antiviral activity against HCoV-OC43 in RD cells compared with the enzymatic assay results. Mpro and PLpro, as essential proteases for processing viral polyproteins, were proposed as targets for anti-SARS-CoV-2 drug discovery soon after the outbreak, as well as in the previous SARS-CoV and MERS-CoV pandemics [45–49]. Their conservation could be observed in many coronaviruses when we performed protein BLAST searches in the UniProt database. For SARS-CoV-2 Mpro, the BLAST resulted in 135 hits (identity ≥ 30% and E-value ≤ e^−5^), covering all four genera in the *Orthocoronavirinae* subfamily (*Alphacoronavirus*, *Betacoronavirus*, *Gammacoronavirus*, and *Deltacoronavirus*) (Supplementary Fig.S10D). For SARS-CoV-2 PLpro, the BLAST resulted in 51 hits (identity ≥ 30% and E-value ≤ e^−5^), mostly clustered in the *Betacoronavirus* genus but covering all its subgenera (Supplementary Fig.S10E).

Regarding the druggability of the two compounds, additional analysis was conducted using the website ADMETlab 3.0 (https://admetlab3.scbdd.com/) [50]. Both compounds were determined to possess good synthetic accessibility and met the criteria of the Lipinski, Pfizer, GSK, and GoldenTriangle rules, indicating an overall favorable ADMET profile. They demonstrated excellent stability against human liver microsomes (see Supporting information 2) but exhibited poor absorption and distribution, moderate clearance, and a miscellaneous toxicity profile. These findings highlight the need for further optimization in the hit-to-lead process. Considering the interaction between sydowiol B and Mpro/PLpro, it is recommended to incorporate positively charged groups at the methyl group (or in close proximity) on the terminal two benzene rings, while negatively charged groups are preferred at the methyl group (or in close proximity) on the middle benzene ring to further enhance affinity. Additionally, due to the high polarity of sydowiol B, the introduction of additional hydrophobic groups is crucial to balance its ADMET properties.

Unlike traditional drug discovery, dual-targeting or multi-targeting inhibitors are not highly restricted to a specific target, which is challenging to pursue. Their dual/multi-targeting feature confers them with increased efficiency, safety, and a higher barrier to resistance. Considering the pathogenesis of most diseases and the massive interaction between pathogens and hosts, the traditional "one drug, one target" strategy faces inevitable challenges [51–53]. Drug repurposing and drug combinations are different ways of dual/multi-targeting strategies. In the past 20 years, research aiming for this strategy has significantly grown. Of course, the promiscuity it possesses cannot be ignored, and the adverse effects of sydowiol B remain to be deeply investigated.

## Conclusion

In summary, we identified two natural products, sydowiol B and violaceol I, as dual inhibitors of SARS-CoV-2 Mpro and PLpro. We further confirmed that the inhibition of sydowiol B was performed by binding at the nano-channel of Mpro that limited the accommodation of substrates, and binding at the active site of PLpro that subsequently induced the instability of the structural Zn^2+^ and might impair the interaction of PLpro with ISG15 and di-ubiquitin. In addition, sydowiol B and violaceol I both exhibited broad-spectrum antiviral activity against three homologous coronaviruses and in vitro inhibition on HCoV-OC43.

## Materials and methods

### Protein production and purification

The sequences encoding SARS-CoV-2 Mpro and PLpro were synthesized by GENEWIZ (Azenta Life Sciences). The SARS-CoV-2 Mpro gene (ORF1ab polyprotein residues 3264–3569, GenBank code: NC_045512) was cloned into the pGEX-6p-1 vector with five additional N-terminal residues (SAVLQ) and eight extra C-terminal residues (GPHHHHHH), as previously reported [30]. The SARS-CoV-2 PLpro gene (ORF1ab polyprotein residues 1564–1878, GenBank code: NC_045512) was cloned into the pET28a-SUMO vector between the BamHI and XhoI restriction sites. Site-directed mutagenesis was performed on the recombinant Mpro and PLpro plasmids using the Trelief® SoSoo Cloning Kit (Beijing Tsingke Biotech Co., Ltd.). Additionally, the homologous Mpro and PLpro genes from SARS-CoV, MERS-CoV, and BtCoV rp3 were synthesized following the same strategy.

For protein expression, the recombinant plasmid was transformed into *E. coli* BL21 (DE3) cells (Weidibio, catalog no. EC1002), which were cultured in Luria Broth medium containing antibiotics at 37 °C until the OD_600_ reached 0.6–0.8. IPTG was then added to a final concentration of 0.4 mM, and cultures were further incubated for 16–20 h at 18 °C. Cell pellets were collected by centrifugation and resuspended in buffer A (20 mM Tris–HCl pH 7.8, 150 mM NaCl, 10 mM imidazole, and 5 mM β-mercaptoethanol). After lysis by sonication and ultracentrifugation at 10,000 g and 4 °C for 1 h, the supernatant was loaded onto a Ni–NTA affinity column (Qiagen). The recombinant protein was purified by washing with 20 mL of buffer A and eluted with 15 mL of buffer B (20 mM Tris–HCl pH 7.8, 150 mM NaCl, 300 mM imidazole, and 5 mM β-mercaptoethanol). The enriched protein was further purified by size-exclusion chromatography (HiLoad™ 16/60 Superdex 75, Cytiva) and stored in 20 mM Tris–HCl pH 7.8, 150 mM NaCl, 5 mM β-mercaptoethanol, and 20% v/v glycerol. For PLpro purification, an additional step was performed after elution with buffer B, where the protein was cleaved by the SUMO protease (ULP1) to remove the N-terminal SUMO tag before loading onto the size-exclusion chromatography column.

### Enzymatic activity assay

Fluorescence resonance energy transfer (FRET) assays were employed using two fluorescent substrates synthesized by NJPeptide: MCA-AVLQSGFR-Lys(Dnp)-Lys-NH2 and Z-RLRGG-AMC, corresponding to the recognition sequences of Mpro and PLpro proteases, respectively. Upon cleavage, the fluorescence intensity of the substrates could be monitored at 405 nm (excited at 320 nm) and 460 nm (excited at 360 nm), respectively.

In the high-throughput screening, 200 nM of Mpro or PLpro was mixed with 40 μM of compounds or DMSO (negative control) and incubated for 30 min at room temperature. The substrate was pipetted into a black 96-well plate with 1 μL per well, and the 49 μL incubated mixture was added to initiate the reaction, which was monitored for 30 min at 37 °C. The remaining enzymatic activity of Mpro or PLpro was proportional to the reaction rate. The final solution contained 200 nM Mpro or PLpro, 40 μM compound or DMSO, 30 μM substrate, 50 mM Tris–HCl pH 7.8, 100 mM NaCl, 1 mg/mL BSA, and 0.01% v/v Triton X-100. For IC_50_ measurement, the same condition was employed, but the protease was incubated with a series of gradient-diluted compounds or DMSO (negative control).

### Thermal shift assay (TSA)

The purified protein was diluted to 0.5 mg/mL with 20 mM Tris–HCl pH 7.8 and 150 mM NaCl, then incubated with compounds or DMSO for 30 min at room temperature. The reaction was conducted in 384-well plates, with 10 μL per well containing 9 μL of the mixed solution and 1 μL of SYPRO™ Orange Protein Gel Stain (Invitrogen). After centrifugation, the melting curve was monitored using an Applied Biosystems® QuantStudio™ 6 Pro Real-Time PCR System (Thermo Fisher Scientific). The first step involved incubation at 25 °C for 10 min, followed by gradient heating from 25 °C to 95 °C at a rate of 0.075 °C/s. T_m_ values were fitted and calculated using the Quantstudio™ Design & Analysis Software v1.5.2. The experiment was performed in triplicate.

### Surface plasmon resonance (SPR) assay

The affinity of SARS-CoV-2 Mpro/PLpro and their homologs with compounds was determined using a Biacore 1 K (Cytiva) instrument. The recombinant protein was immobilized on a series S sensor chip CM5. Association and dissociation were performed using a predefined multi-cycle kinetics/affinity procedure with 8 gradient-diluted concentrations of compounds in a buffer containing 10 mM HEPES pH 7.4, 150 mM NaCl, 3 mM EDTA, 0.005% v/v Surfactant P20, and 5% v/v DMSO. The results were analyzed using a predefined multi-cycle kinetics procedure to calculate the K_a_, K_d_, and K_D_ values.

### Molecular docking

To investigate the possible interaction and binding site of sydowiol B with SARS-CoV-2 Mpro/PLpro and reduce the influence of PDB models on the results, we conducted multiple blind molecular docking simulations using AutoDock Vina. For Mpro, the following PDB models were used: 5R80, 5RE4, 7ALH, 7K0G, 7LMD, 7LZT, 6WTM, 6XHU, 7B3E, 7BB2, 7BE7, 7BGP, 7C2Q, and 7C2Y. For PLpro, the PDB models included 6WUU, 6WZU, 6XA9, 6XAA, 7JIW, 7JRN, 7LBR, 7OFS, 8G62, 7LLF, and 7NT4. The 3D structure of sydowiol B was obtained from the PubChem database and converted into PDB format using the OpenBabel program. Before docking, the ligands from the PDB models were removed, and the grid was set to cover the entire structure of Mpro/PLpro. Each docking run generated 9 conformations of sydowiol B, and the results were analyzed using PyMOL.

### Molecular dynamics (MD) simulations

Molecular dynamics (MD) simulations were performed using the CUDA-accelerated GROMACS-2022.2 program with the AMBER99SB-ILDN force field for the protein and the GAFF force field for the ligand. Ligand topology files were generated using Acpype [54–56]. The complex structure of the protein and the ligand was obtained from molecular docking results and used as the initial coordinate file for the MD simulations. The complex was solvated in a cubic periodic box with the TIP3P water model, and the system's charges were neutralized by adding sodium and chloride ions to achieve a final ionic strength of 0.15 M. Energy minimization was performed for 5000 steps using the steepest descent method to eliminate unfavorable contacts in the system. Subsequently, the canonical (NVT) and isothermal-isobaric (NPT) ensembles were used for equilibration, resulting in a constant temperature of 310 K and pressure of 1 bar for the system. Finally, a 100 ns MD simulation was carried out for the equilibrated system. Subsequent MD analyses were performed using GROMACS built-in modules, and the data was processed using Origin 2021, PyMOL, and VMD.

Free energy and decomposition analyses were performed using the gmx_MMPBSA program [57]. Frames were selected starting from the equilibrium of the system, with an interval of 20 ps. Other parameters were kept as default. The volume of pockets was calculated using the SiteMap module (Evaluate a single binding site region) of the Schrödinger software. The structural communication within the complex was analyzed by the WebPSN server [58], utilizing the lowest energy conformation obtained from principal component analysis (PCA).

### Antiviral and cytotoxicity assays

For the antiviral assay, RD cells (Procell, catalog no. CL-0193) were seeded at a density of 5 × 10^4^ cells/well in 48-well plates one day prior to the experiment. Gradient-diluted compounds or DMSO were added to preincubate the cells for 1 h, followed by infection with viruses at a multiplicity of infection (MOI) of 0.01. The cells were then incubated for an additional 48 h. The supernatant was subjected to qRT-PCR analysis, while the cells were used for an immunofluorescence assay. Viral genomes were extracted using an Automatic Nucleic Acid Extraction Instrument (Vazyme) and the Virus DNA/RNA Extraction Kit 2.0 (Vazyme). Genome copies were quantified using an Applied Biosystems® QuantStudio™ 6 Pro Real-Time PCR System (Thermo Fisher Scientific) and the HiScript II One Step qRT-PCR SYBR Green Kit (Vazyme). The infected cells were fixed with 8% formalin overnight, blocked with 5% milk and 0.2% Triton X-100 in PBS for 1 h, and then detected using a rabbit anti-OC43 nucleocapsid primary antibody (ABclonal), a CoraLite488-conjugated Goat Anti-Rabbit lgG(H + L) (Proteintech), and the DAPI solution (ready-to-use) (ACMEC). Images were acquired and merged using the Cytation3 Imaging System (BioTek).

For the cytotoxicity assay, RD cells were seeded at a density of 2 × 10^4^ cells/well in 96-well plates one day prior to the experiment. Gradient-diluted compounds or DMSO were added, and the cells were incubated for 48 h. Subsequently, the cells were subjected to a CCK8 assay (Beyotime) according to the manufacturer's instructions. All experiments were performed in triplicate, and the data were fitted to curves using GraphPad Prism.

### Statistical analysis

Data were analyzed and visualized using GraphPad Prism 8.0 (San Diego, CA, USA) or IBM SPSS Statistics 23 (Chicago, IL, USA). Results are presented as mean ± standard deviation (SD). Statistical variances were assessed using one-way ANOVA and significance levels were defined as follows: ^*^P < 0.05, ^**^P < 0.01, ^***^P < 0.001, and ^****^P < 0.0001, respectively.

## Supplementary Information

Supplementary Material 1.

Supplementary Material 2.

## Data Availability

Data will be made available on request.
