# Supplementary material for: Unveiling the mechanism of action of a novel natural dual inhibitor of SARS-CoV-2 Mpro and PLpro with molecular dynamics simulations
Source: Nat Prod Bioprospect. 2025 Jan 4;15(1):3. doi: 10.1007/s13659-024-00486-4 (PMC11699025; doi:10.1007/s13659-024-00486-4)
Supplement: Supplementary file 1 — Supplementary Material 1. [file 13659_2024_486_MOESM1_ESM.docx]

Supporting Information for

**ORIGINAL ARTICLE**

**Unveiling the mechanism of action of a novel natural dual inhibitor of SARS-CoV-2 Mpro and PLpro with molecular dynamics simulations**

Xiaoxia Gu^†^, Xiaotian Zhang^†^, Xueke Zhang, Xinyu Wang, Weiguang Sun*, Yonghui Zhang* and Zhengxi Hu*

*Corresponding Authors.

E-mail addresses: weiguang_sun@hust.edu.cn (Weiguang Sun), zhangyh@mails.tjmu.edu.cn (Yonghui Zhang), and hzx616@126.com (Zhengxi Hu).

^†^These authors contributed equally to this work.

**Table S1** The effect of sydowiol B on the thermal stability of SARS-CoV-2 Mpro wild type and mutants.

| **T_m_ value (℃)** | **Sydowiol B** | | | |  |
| --- | --- | --- | --- | --- | --- |
|  | **100 µM (△T_m_)** | **50 µM (△T_m_)** | **25 µM (△T_m_)** | **0 µM** | **△T_m_ （relative to WT）** |
| WT | 56.19 ± 0.14 (1.19) | 55.99 ± 0.15 (0.99) | 55.84 ± 0.43 (0.84) | 55.00 ± 0.12 |  |
| F3A | 53.05 ± 0.12 (1.05) | 52.84 ± 0.19 (0.84) | 53.05 ± 0.03 (1.05) | 52.00 ± 0.09 | -3.00 |
| R4A | 54.54 ± 0.06 (0.84) | 54.25 ± 0.27 (0.55) | 54.16 ± 0.03 (0.46) | 53.70 ± 0.15 | -1.30 |
| K5A | 48.38 ± 0.03 (1.03) | 48.41 ± 0.03 (1.06) | 47.98 ± 0.07 (0.63) | 47.35 ± 0.25 | -7.65 |
| H41A | 48.26 ± 0.20 (0.31) | 48.68 ± 0.20 (0.73) | 48.86 ± 0.15 (0.91) | 47.95 ± 0.12 | -7.05 |
| Y126A | 48.14 ± 0.06 (1.84) | 48.19 ± 0.09 (1.89) | 47.88 ± 0.22 (1.58) | 46.30 ± 0.30 | -8.70 |
| N142A | 58.18 ± 0.03 (5.56) | 57.22 ± 0.07 (4.60) | 56.43 ± 0.24 (3.81) | 52.62 ± 0.15 | -2.38 |
| G143A | 48.86 ± 0.06 (1.58) | 49.12 ± 0.09 (1.84) | 49.08 ± 0.01 (1.80) | 47.28 ± 0.06 | -7.72 |
| C145A | 53.34 ± 0.46 (0.78) | 53.62 ± 0.00 (1.06) | 53.34 ± 0.34 (0.78) | 52.56 ± 0.12 | -2.44 |
| M165A | 49.53 ± 0.03 (0.96) | 49.58 ± 0.06 (1.01) | 49.36 ± 0.06 (0.79) | 48.57 ± 0.01 | -6.43 |
| E288A | 54.03 ± 0.10 (1.14) | 53.79 ± 0.19 (0.90) | 53.21 ± 0.11 (0.32) | 52.89 ± 0.18 | -2.11 |
| F291A | 61.29 ± 0.01 (-0.08) | 61.37 ± 0.01 (0.00) | 61.44 ± 0.01 (0.07) | 61.37 ± 0.12 | 6.37 |

**Table S2** The binding energy between two chains of Mpro in apo or com state, and between sydowiol B and Mpro, decomposed by its contributions (calculated by gmx_MMPBSA).

|  | **Apo** | **Com** | |
| --- | --- | --- | --- |
| Receptor_ligand | chain A_chain B | chain A_chain B | Mpro_sydowiol B |
| Delta Energy Component  (Complex - Receptor - Ligand) | Average ± SEM (kcal/mol) | Average ± SEM (kcal/mol) | Average ± SEM (kcal/mol) |
| ΔBOND | 0.00 ± 0.00 | -0.00 ± 0.00 | 0.00 ± 0.00 |
| ΔANGLE | 0.00 ± 0.00 | -0.00 ± 0.00 | -0.00 ± 0.00 |
| ΔDIHED | 0.00 ± 0.00 | 0.00 ± 0.00 | 0.00 ± 0.00 |
| ΔVDWAALS | -179.43 ± 0.12 | -175.88 ± 0.11 | -39.26 ± 0.07 |
| ΔEEL | -356.46 ± 0.53 | -306.45 ± 0.42 | -46.96 ± 0.22 |
| Δ1-4 VDW | -0.00 ± 0.00 | -0.00 ± 0.00 | 0.00 ± 0.00 |
| Δ1-4 EEL | -0.00 ± 0.00 | -0.00 ± 0.00 | 0.00 ± 0.00 |
| ΔEGB | 426.15 ± 0.43 | 389.14 ± 0.36 | 61.02 ± 0.11 |
| ΔESURF | -24.16 ± 0.01 | -22.70 ± 0.01 | -5.41 ± 0.01 |
| ΔGGAS | -535.89 ± 0.50 | -482.33 ± 0.43 | -86.22 ± 0.17 |
| ΔGSOLV | 401.99 ± 0.43 | 366.44 ± 0.36 | 55.61 ± 0.11 |
| ΔTOTAL | -133.89 ± 0.14 | -115.89 ± 0.13 | -30.60 ± 0.09 |

**Table S3** The effect of sydowiol B on the thermal stability of SARS-CoV-2 PLpro wild type and mutants.

| **T_m_ value (℃)** | **Sydowiol B** | | | |  |
| --- | --- | --- | --- | --- | --- |
|  | **100 µM (△T_m_)** | **50 µM (△T_m_)** | **25 µM (△T_m_)** | **0 µM** | **△T_m_ (relative to WT)** |
| WT | 51.24 ± 0.36 (5.29) | 50.31 ± 0.06 (4.36) | 49.79 ± 0.43 (3.84) | 45.95 ± 0.30 |  |
| D164A | 44.58 ± 0.62 (2.03) | 45.84 ± 0.11 (3.29) | 46.83 ± 0.21 (4.28) | 42.55 ± 0.25 | -3.40 |
| R166A | 48.83 ± 0.61 (1.15) | 50.86 ± 0.55 (3.18) | 50.01 ± 0.65 (2.33) | 47.68 ± 0.27 | 1.73 |
| Y264A | 46.21 ± 0.86 (0.01) | 45.93 ± 0.03 (-0.27) | 46.48 ± 0.43 (0.28) | 46.20 ± 0.03 | 0.25 |
| Y268A | 50.46 ± 0.65 (3.82) | 48.51 ± 0.22 (1.87) | 49.74 ± 0.16 (3.10) | 46.64 ± 0.08 | 0.69 |
| Y273A | 51.09 ± 0.23 (-0.85) | 50.59 ± 0.10 (-1.35) | 51.21 ± 0.00 (-0.73) | 51.94 ± 0.15 | 5.99 |
| E214A | 48.71 ± 0.35 (2.88) | 49.41 ± 0.51 (3.58) | 48.88 ± 0.34 (3.05) | 45.83 ± 0.31 | -0.12 |
| Y251A | 49.13 ± 0.66 (3.76) | 47.78 ± 0.39 (2.41) | 46.46 ± 0.07 (1.09) | 45.37 ± 0.29 | -0.58 |
| E252A | 44.10 ± 0.20 (0.75) | 45.88 ± 0.48 (2.53) | 47.86 ± 0.13 (4.51) | 43.35 ± 0.13 | -2.60 |
| L253A | 48.43 ± 0.31 | 49.01 ± 0.51 | 50.09 ± 0.49 | - |  |
| K254A | 48.33 ± 0.52 (5.03) | 46.61 ± 0.16 (3.31) | 46.23 ± 0.29 (2.93) | 43.30 ± 0.44 | -2.65 |
| T257A | 48.53 ± 0.25 (4.93) | 48.33 ± 0.21 (4.73) | 47.76 ± 0.27 (4.16) | 43.60 ± 0.28 | -2.35 |
| F258A | 49.73 ± 0.43 | 48.41 ± 0.23 | 47.68 ± 0.06 | - |  |
| V303A | 52.24 ± 0.13 (5.12) | 49.52 ± 0.19 (2.40) | 48.31 ± 0.25 (1.19) | 47.12 ± 0.11 | 1.17 |

**Table S4** The binding energy between sydowiol B and PLpro, decomposed by its contributions (calculated by gmx_MMPBSA).

|  | **Compound** |
| --- | --- |
| Receptor_ligand | PLpro_sydowiol B |
| Delta Energy Component  (Complex - Receptor - Ligand) | Average ± SEM (kcal/mol) |
| ΔBOND | -0.00 ± 0.00 |
| ΔANGLE | 0.00 ± 0.00 |
| ΔDIHED | 0.00 ± 0.00 |
| ΔVDWAALS | -29.95 ± 0.05 |
| ΔEEL | -24.51 ± 0.17 |
| Δ1-4 VDW | 0.00 ± 0.00 |
| Δ1-4 EEL | -0.00 ± 0.00 |
| ΔEGB | 33.15 ± 0.14 |
| ΔESURF | -3.98 ± 0.01 |
| ΔGGAS | -54.46 ± 0.17 |
| ΔGSOLV | 29.18 ± 0.14 |
| ΔTOTAL | -25.29 ± 0.07 |

**Table S5** The thermal stability of Mpro from homologous coronaviruses and the effects of sydowiol B on their stability.

| **T_m_ value (℃)** | **Sydowiol B** | | | |  |
| --- | --- | --- | --- | --- | --- |
|  | **100 µM (△T_m_)** | **50 µM (△T_m_)** | **25 µM (△T_m_)** | **0 µM** | **△T_m_ （relative to SARS-CoV-2 Mpro）** |
| SARS-CoV Mpro | 57.17 ± 0.19 (1.25) | 56.82 ± 0.25 (0.90) | 56.37 ± 0.28 (0.45) | 55.92 ± 0.20 | 0.16 |
| MERS-CoV Mpro | 47.47 ± 0.33 (1.37) | 47.33 ± 0.00 (1.23) | 47.27 ± 0.16 (1.17) | 46.10 ± 0.15 | -9.66 |
| BtCoV Rp3 Mpro | 46.66 ± 0.25 (0.91) | 46.93 ± 0.13 (1.18) | 46.81 ± 0.53 (1.06) | 45.75 ± 0.03 | -10.01 |

**Table S6** The binding affinity of compounds with Mpro from four coronaviruses.

| **Compound** | **SARS-CoV-2 Mpro** | | | **SARS-CoV Mpro** | | | **MERS-CoV Mpro** | | | **BtCoV Rp3 Mpro** | | |
| --- | --- | --- | --- | --- | --- | --- | --- | --- | --- | --- | --- | --- |
|  | **k_a_ (1/Ms)** | **k_d_ (1/s)** | **K_D_ (M)** | **k_a_ (1/Ms)** | **k_d_ (1/s)** | **K_D_ (M)** | **k_a_ (1/Ms)** | **k_d_ (1/s)** | **K_D_ (M)** | **k_a_ (1/Ms)** | **k_d_ (1/s)** | **K_D_ (M)** |
| GC376 | 2.73e^+03^ | 1.62e^-02^ | 5.93e^-06^ | 4.32e^+03^ | 6.53e^-06^ | 1.51e^-09^ | 3.15e^+03^ | 1.71e^-06^ | 5.44e^-10^ | 2.64e^+03^ | 6.34e^-03^ | 2.40e^-06^ |
| Sydowiol B | 2.52e^+02^ | 6.63e^-03^ | 2.63e^-05^ | 3.27e^+02^ | 8.28e^-04^ | 2.54e^-06^ | 6.72e^+02^ | 6.29e^-03^ | 9.35e^-06^ | 2.86e^+02^ | 5.52e^-04^ | 1.93e^-06^ |
| Violaceol I | 9.69e^+02^ | 1.99e^-03^ | 2.06e^-06^ | 5.47e^+02^ | 4.57e^-04^ | 8.35e^-07^ | 4.54e^+02^ | 1.39e^-02^ | 3.06e^-05^ | 5.32e^+02^ | 1.03e^-04^ | 1.93e^-07^ |

**Table S7** The thermal stability of PLpro from homologous coronaviruses and the effects of sydowiol B on their stability.

| **T_m_ value (℃)** | **Sydowiol B** | | | |  |
| --- | --- | --- | --- | --- | --- |
|  | **100 µM (△T_m_)** | **50 µM (△T_m_)** | **25 µM (△T_m_)** | **0 µM** | **△T_m_ （relative to SARS-CoV-2 PLpro）** |
| SARS-CoV PLpro | 53.82 ± 0.03 (3.68) | 52.84 ± 0.14 (2.70) | 52.79 ± 0.00 (2.65) | 50.14 ± 0.10 | 2.56 |
| MERS-CoV PLpro | 57.67 ± 0.32 (2.78) | 57.20 ± 0.13 (2.31) | 56.95 ± 0.13 (2.06) | 54.89 ± 0.21 | 7.31 |
| BtCoV Rp3 PLpro | 53.59 ± 0.20 (4.00) | 53.87 ± 0.13 (4.28) | 52.79 ± 0.27 (3.20) | 49.59 ± 0.25 | 2.01 |

**Table S8** The binding affinity of compounds with PLpro from four coronaviruses.

| **Compound** | **SARS-CoV-2 PLpro** | | | **SARS-CoV PLpro** | | | **MERS-CoV PLpro** | | | **BtCoV Rp3 PLpro** | | |
| --- | --- | --- | --- | --- | --- | --- | --- | --- | --- | --- | --- | --- |
|  | **k_a_ (1/Ms)** | **k_d_ (1/s)** | **K_D_ (M)** | **k_a_ (1/Ms)** | **k_d_ (1/s)** | **K_D_ (M)** | **k_a_ (1/Ms)** | **k_d_ (1/s)** | **K_D_ (M)** | **k_a_ (1/Ms)** | **k_d_ (1/s)** | **K_D_ (M)** |
| GRL0617 | 3.68e^+03^ | 8.15e^-01^ | 2.21e^-04^ | 2.07e^+04^ | 5.80e^-01^ | 2.80e^-05^ | 1.13e^+02^ | 3.72e^-02^ | 3.29e^-04^ | 2.69e^+04^ | 5.88e^-01^ | 2.19e^-05^ |
| Sydowiol B | 3.01e^+02^ | 5.71e^-03^ | 1.89e^-05^ | 2.67e^+02^ | 3.78e^-03^ | 1.42e^-05^ | 1.63e^+02^ | 7.33e^-05^ | 4.51e^-07^ | 2.76e^+02^ | 3.26e^-03^ | 1.18e^-05^ |
| Violaceol I | 4.94e^+02^ | 4.63e^-03^ | 9.36e^-06^ | 5.16e^+02^ | 3.88e^-03^ | 7.53e^-06^ | 4.79e^+02^ | 1.59e^-04^ | 3.32e^-07^ | 5.51e^+02^ | 2.90e^-03^ | 5.26e^-06^ |

**Table S9** The *in vitro* antiviral activity of sydowiol B and the analogue violaceol I.

| **Compound** | **HCoV-OC43**  **(in RD cells)** | | |
| --- | --- | --- | --- |
|  | **EC_50_ (µM)** | **CC_50_ (µM)** | **Selection Index (SI)** |
| Violaceol I | 2.38 ± 0.02 | ＞100 | ＞42.02 |
| Sydowiol B | 0.69 ± 0.10 | ＞200 | ＞289.86 |


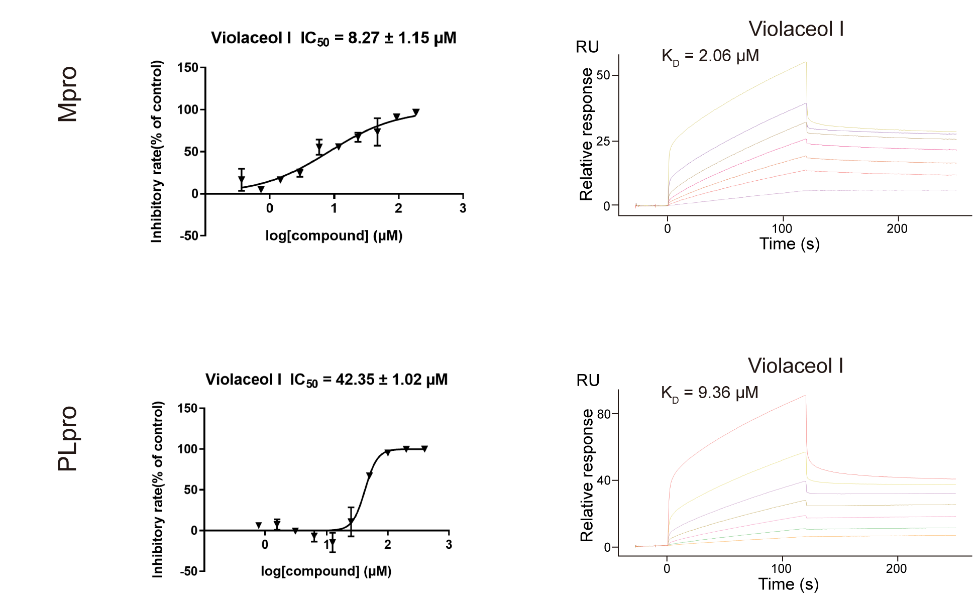


**Fig. S1** The inhibitory activity and binding affinity of violaceol I against SARS-CoV-2 Mpro and PLpro.


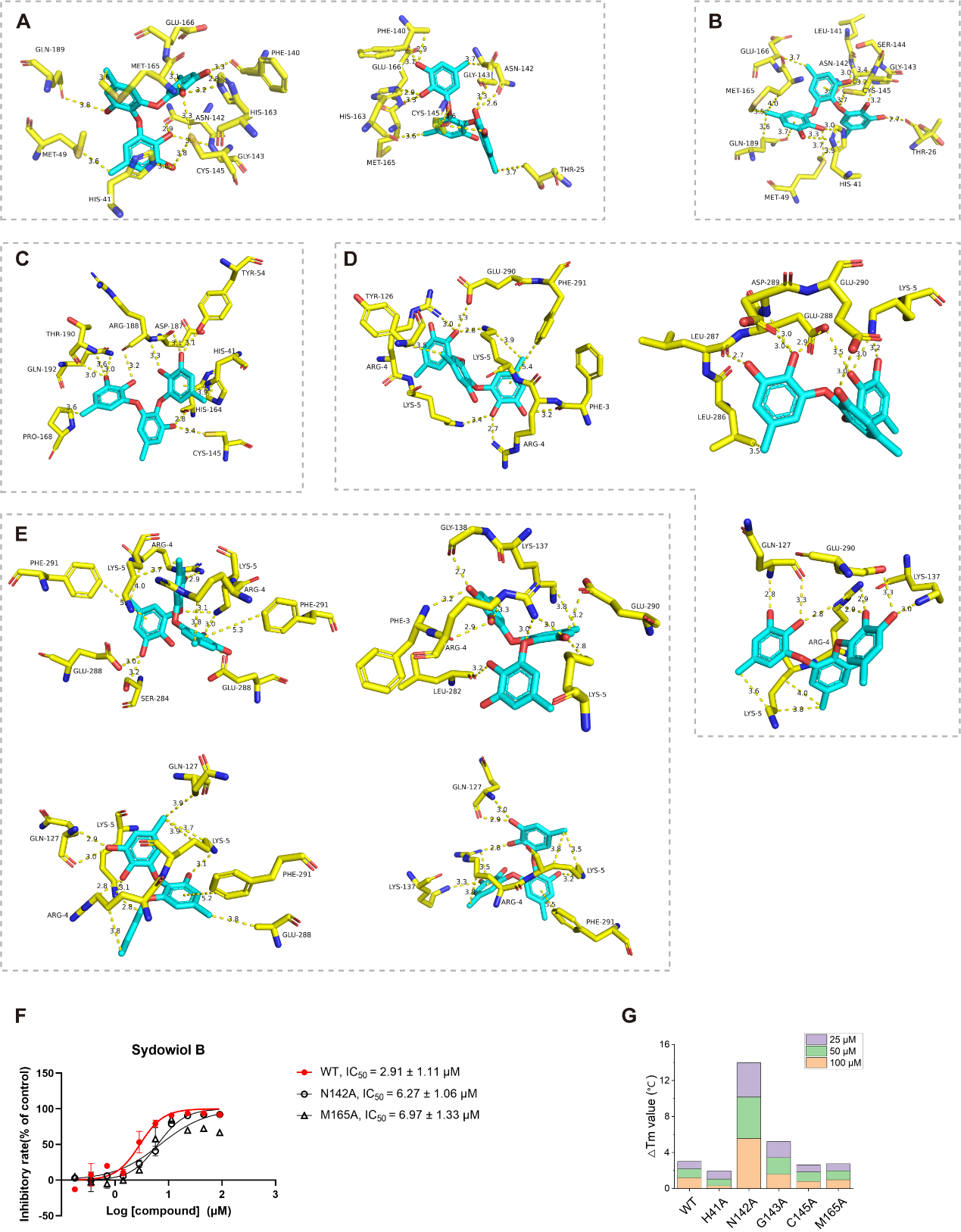


**Fig.** **S2** Molecular docking of sydowiol B with SARS-CoV-2 Mpro. **A-C** Analyses of the interactions between sydowiol B and the active site of Mpro from different docking models: (**A**) Mono_group 1, with models 5RE4 and 7LMD, (**B**) Mono_group 2, with model 7K0G, and (**C**) Mono_group 3, with model 5R80. **D-E** Analyses of the interactions between sydowiol B and the nano-channel of Mpro from different docking models: (**D**) Dimer_group 1, with models 6XHU, 7BGP, and 7C2Y, and (**E**) Dimer_group 2, with models 6WTM, 7BB2, 7C2Q, and 7C2Y. **F** The inhibitory activity of sydowiol B against Mpro mutants derived from the active site residues. **G** The effect of sydowiol B on the thermal stability of Mpro mutants derived from the active site residues.


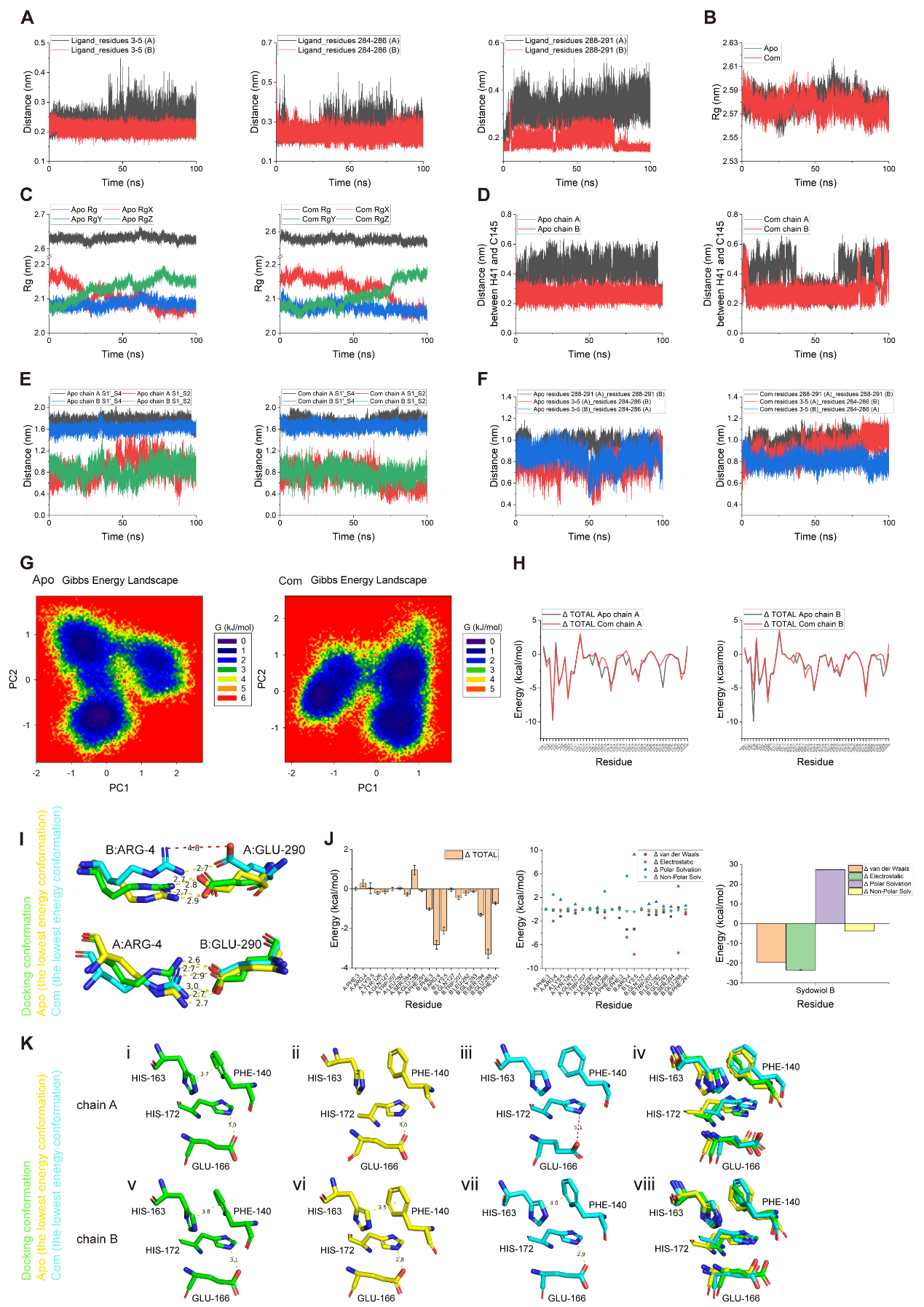


**Fig.** **S3** Sydowiol B bound to SARS-CoV-2 Mpro at the nano-channel. **A** Distances between sydowiol B and residues 3-5 (chain A), residues 3-5 (chain B), residues 284-286 (chain A), residues 284-286 (chain B), residues 288-291 (chain A), and residues 288-291 (chain B) during the simulation. **B** The Rg plot of Mpro in the apo and com (complexed with sydowiol B) states. **C** The decomposition of Rg into three independent axes in the apo and com states. **D** The distance between His41 and Cys145 in the apo and com states. **E** Distances between the S1' loop and S4 loop, and between the S1 loop and S2 helix in the apo and com states. **F** Distances between residues 288-291 (chain A) and 288-291 (chain B), between residues 3-5 (chain A) and 284-286 (chain B), and between residues 3-5 (chain B) and 284-286 (chain A) in the apo and com states. **G** The Gibbs energy landscape of Mpro in the apo and com states. **H** The binding energy between the two chains of Mpro contributed by residues in the dimer interface in the apo and com states. **I** The interaction between Arg4 (chain A/B) and Glu290 (chain B/A) in the docking conformation, apo, and com states (the latter two represent the lowest energy conformations from PCA). **J** The binding energy between Mpro and sydowiol B contributed by individual residues and their decomposition, as well as the decomposition of binding energy from sydowiol B. **K** Conformational changes of the structural markers on protomer activity among the docking conformation, apo, and com states (the latter two represent the lowest energy conformations from PCA).


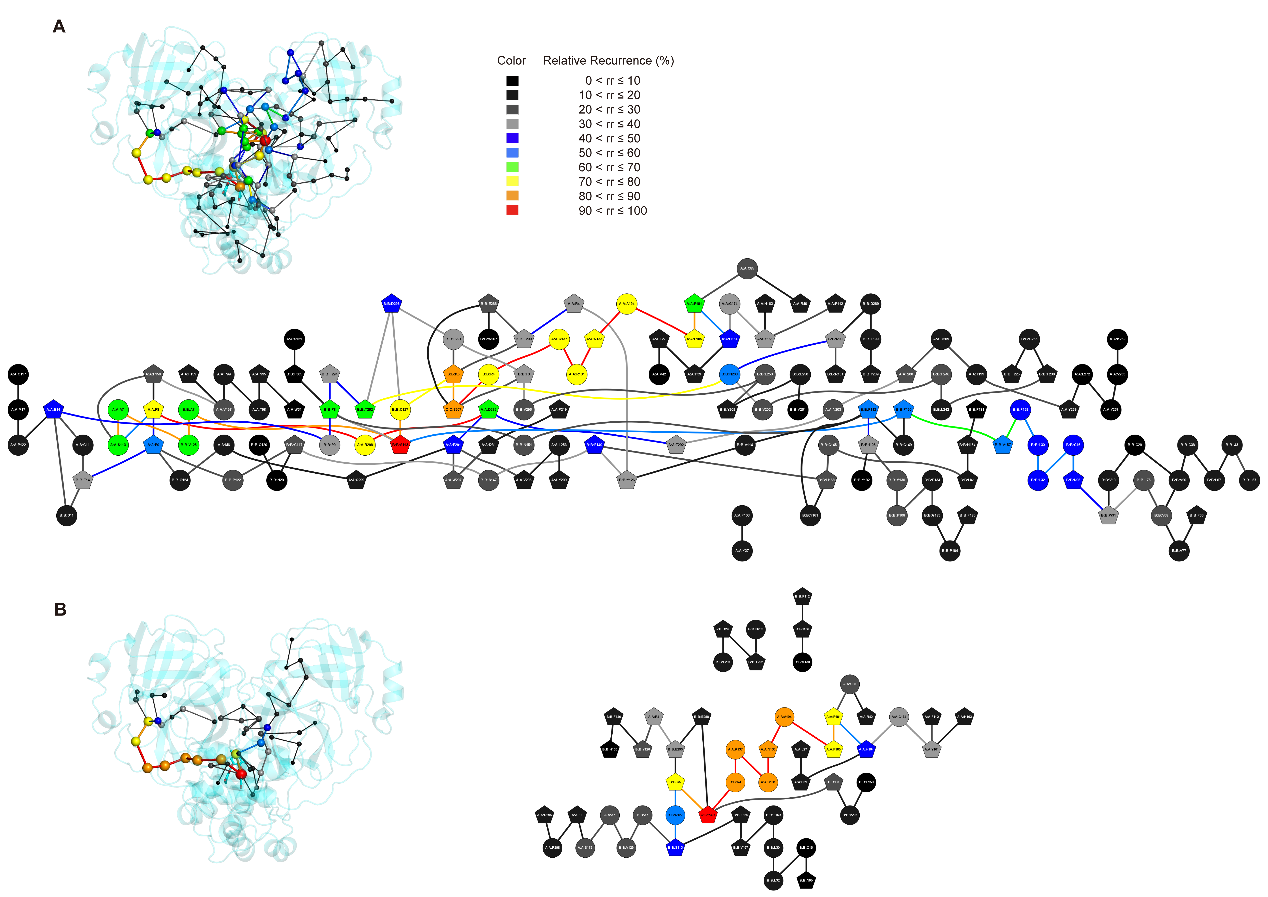


**Fig.** **S4** The PSN analysis of sydowiol B-bound Mpro (C:C:?307 represents sydowiol B, where "?" indicates any non-standard amino acid or nucleotide residue. The node is represented as a sphere centered on the Cα carbon atom of standard amino acids, on the N1 atom of standard nucleotides and on the atom nearest to the geometric center for all other molecules present in the analyzed structure.) **A** The global metapath within the complex, with the color legend indicating the relative recurrence (%) of each path. **B** The filtered metapath within the complex, obtained by applying the requirement "midway through the ligand".


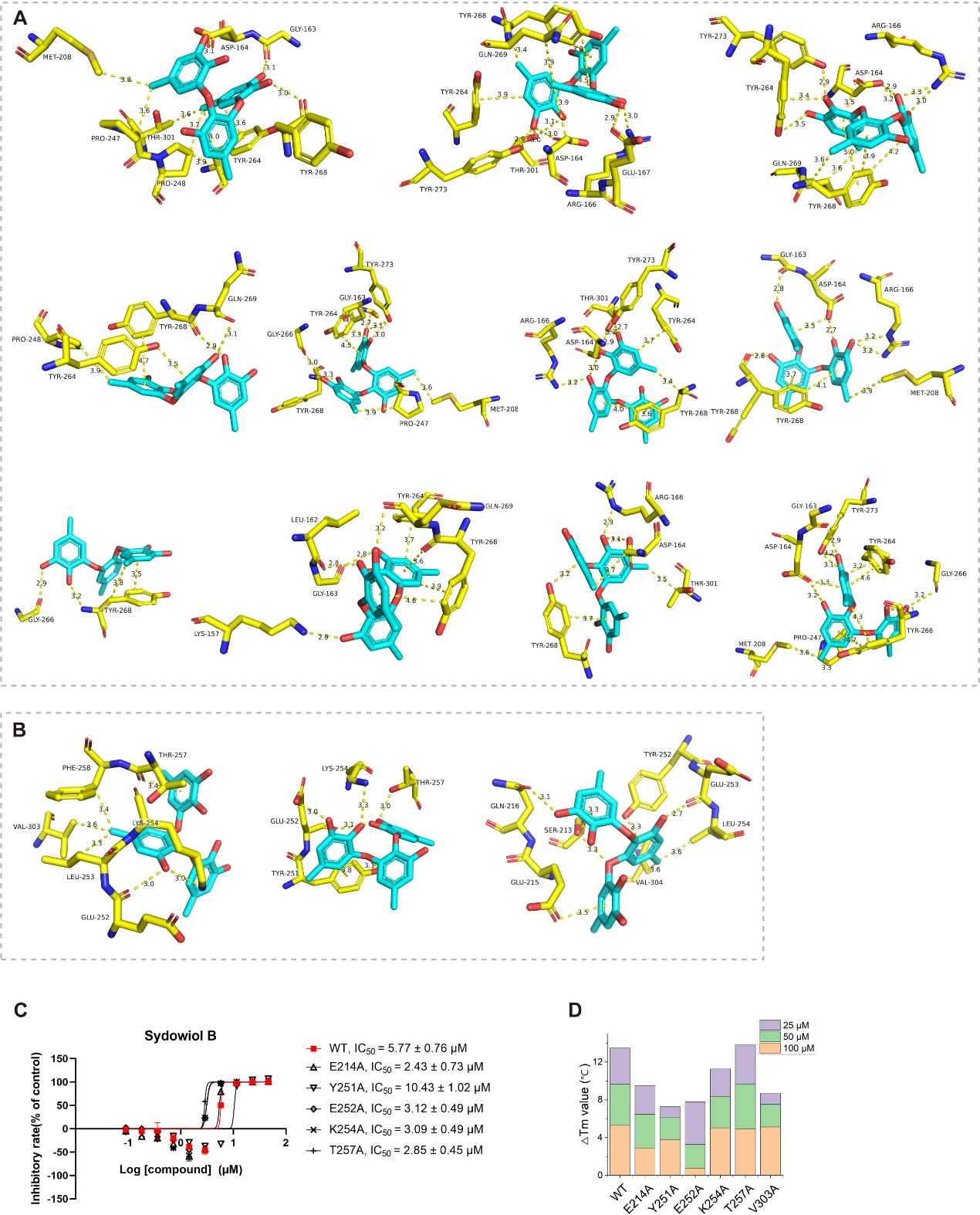


**Fig.** **S5** Docking results of sydowiol B with multiple PDB models of SARS-CoV-2 PLpro. **A** Analysis of the interactions between sydowiol B and the active site of PLpro using PDB models 6WUU, 6WZU, 6XA9, 6XAA, 7JIW, 7JRN, 7JRN, 7LBR, 7OFS, 7OFS, and 8G62. **B** Analysis of the interactions between sydowiol B and the allosteric site of PLpro using PDB models 7LBR, 7LLF, and 7NT4. **C** The inhibitory activity of sydowiol B against PLpro mutants derived from the allosteric site residues. **D** The effect of sydowiol B on the thermal stability of PLpro mutants derived from the allosteric site residues.


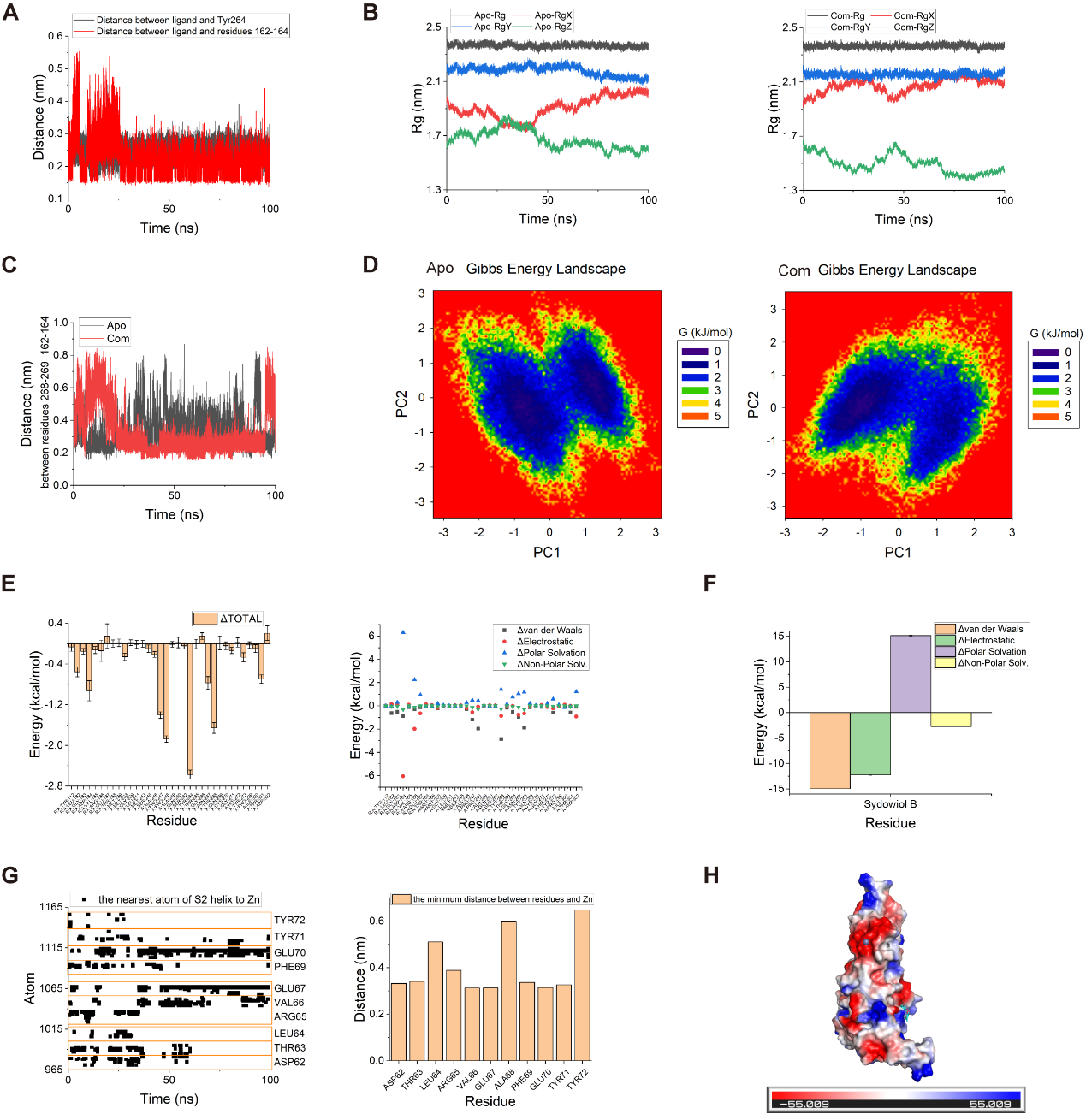


**Fig.** **S6** Sydowiol B bound to SARS-CoV-2 PLpro at the active site. **A** Distances between sydowiol B and residue Tyr264 or residues 162-164 during the simulation. **B** The decomposition of Rg into three independent axes in the apo and com (complexed with sydowiol B) states. **C** The distance between the BL2 loop (residues 268-269) and residues 162-164 in the apo and com states. **D** The Gibbs energy landscape of PLpro in the apo and com states. **E** The binding energy between PLpro and sydowiol B contributed by individual residues and their decomposition. **F** The binding energy between PLpro and sydowiol B contributed by sydowiol B and its decomposition. **G** The nearest atom and the shortest distance between sydowiol B and residues from the S2 helix during the MD simulation. **H** The vacuum electrostatics of PLpro in the com state.


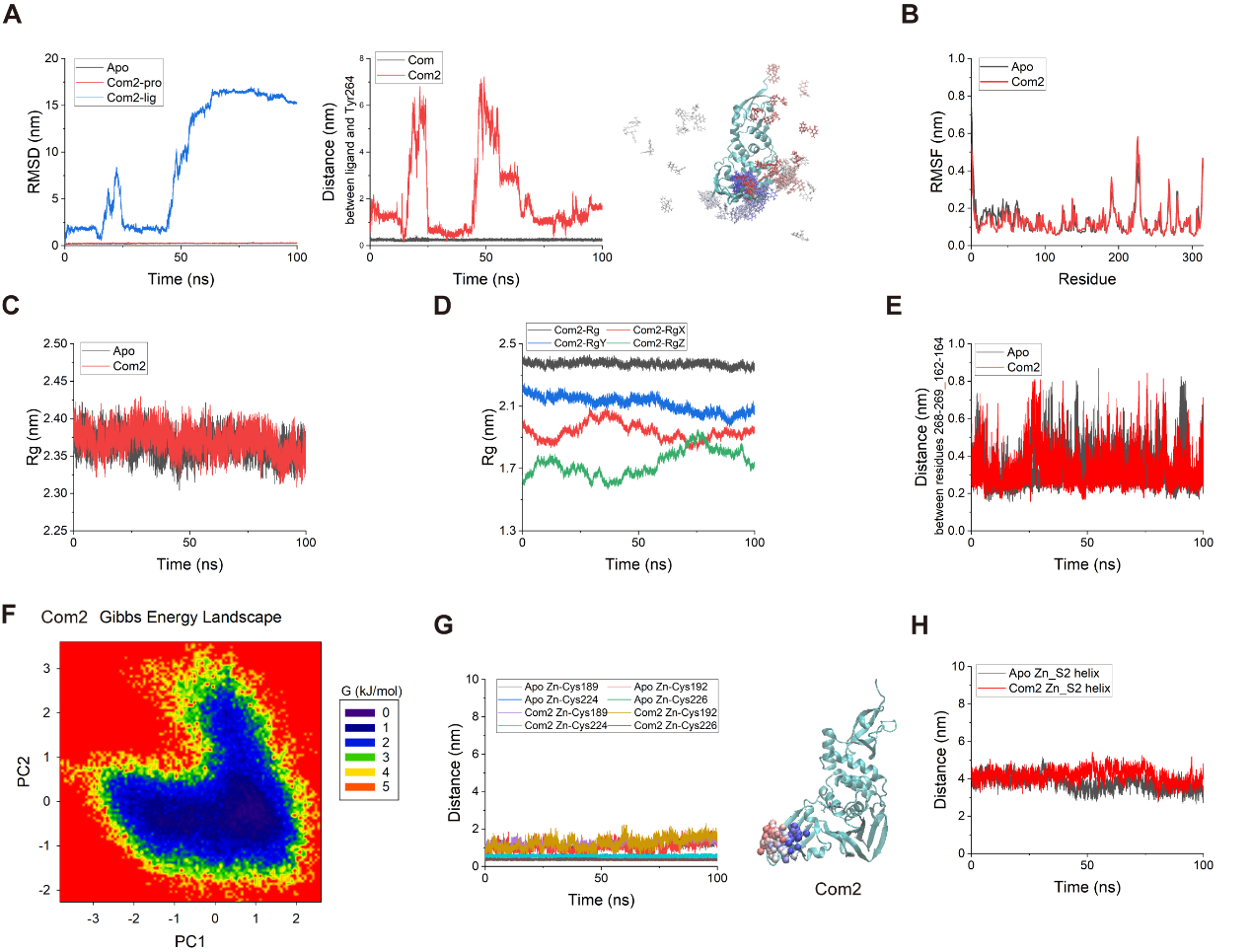


**Fig.** **S7** MD results of PLpro complexed with GRL0617. **A** The RMSD plot of PLpro in the apo (unbound) and com2 (complexed with GRL0617) states, the distance between the ligand and residue Tyr264, and the trajectory of GRL0617 during the simulation after aligning PLpro. **B** The RMSF plot of PLpro in the apo and com2 states. **C** The Rg plot of PLpro in the apo and com2 states. **D** The decomposition of Rg into three independent axes in the com2 state. **E** The distance between the BL2 loop (residues 268-269) and residues 162-164 in the apo and com2 states. **F** The Gibbs energy landscape of PLpro in the com2 state. **G** The distances between the Zn^2+^ ion and the four conserved coordinating cysteines in the apo and com2 states. **H** The distance between the Zn^2+^ ion and the S2 helix in the apo and com2 states.


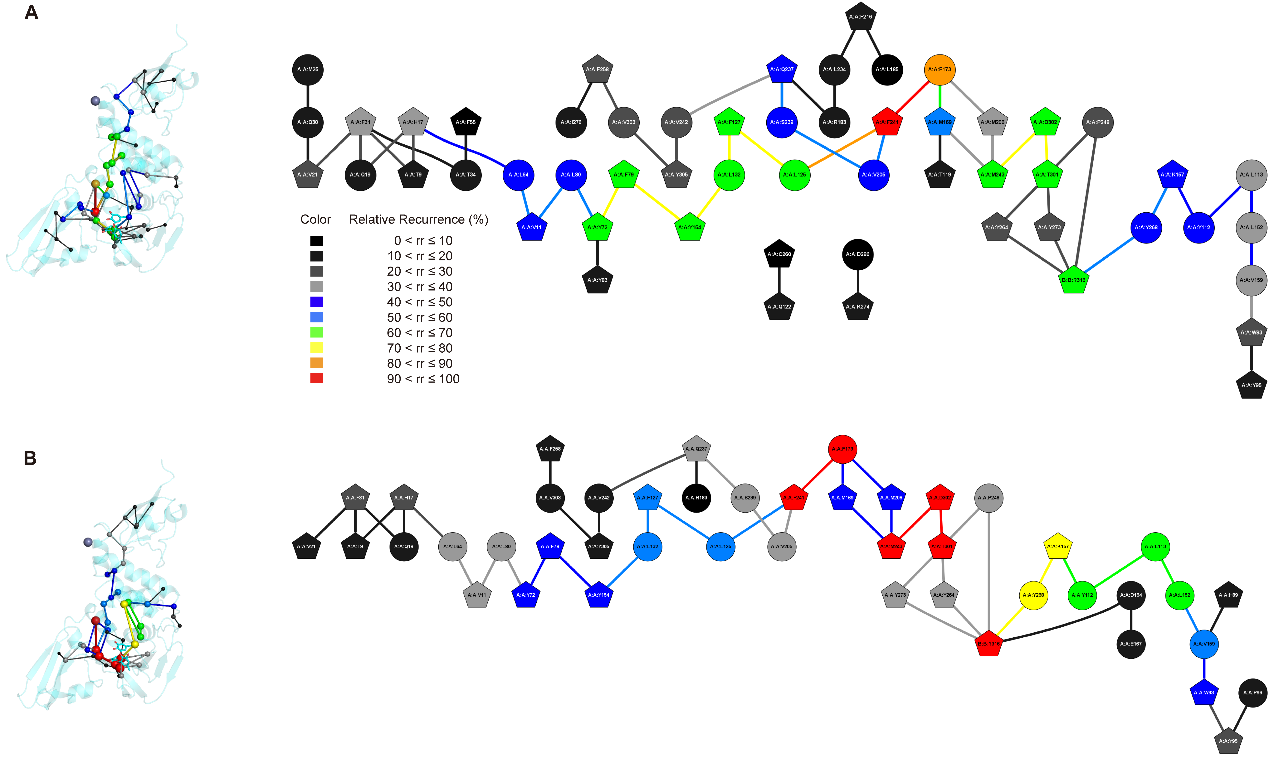


**Fig.** **S8** The PSN analysis of sydowiol B-bound PLpro (B:B:?316 represents sydowiol B, where "?" indicates any non-standard amino acid or nucleotide residue.) (The node is represented as a sphere centered on the Cα carbon atom of standard amino acids, on the N1 atom of standard nucleotides and on the atom nearest to the geometric center for all other molecules present in the analyzed structure.). **A** The global metapath inside the complex and the color legend of relative recurrence (%). **B** The filtered metapath inside the complex by the requirement "midway through the ligand".


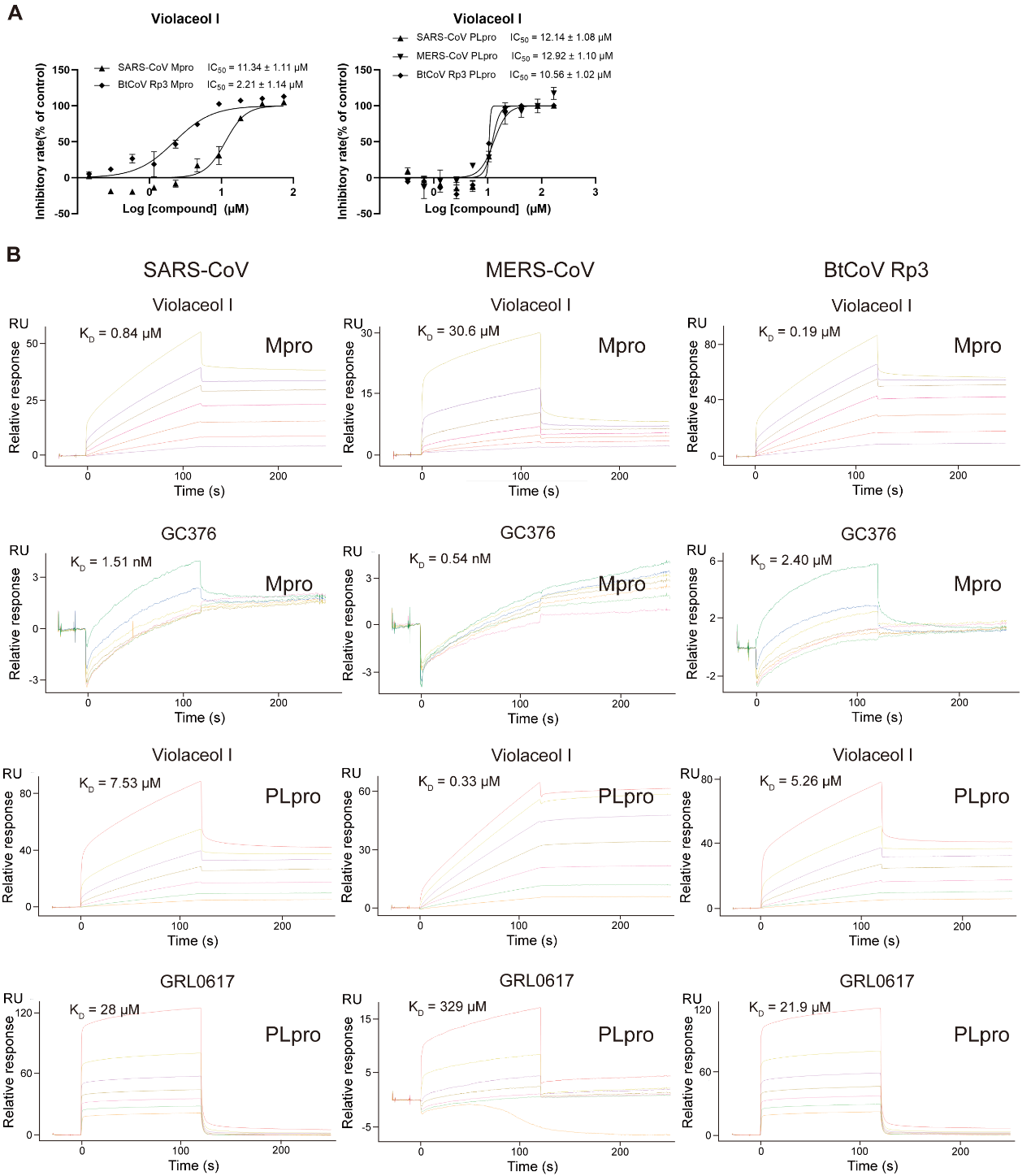


**Fig. S9** The broad-spectrum antiviral activity of violaceol I. **A** The effect of sydowiol B on the enzymatic activity of Mpro and PLpro from homologous coronaviruses. **B** The binding affinity of violaceol I and GC376 with Mpro from homologous coronaviruses, and the binding affinity of violaceol I and GRL0617 with PLpro from the same set of coronaviruses.


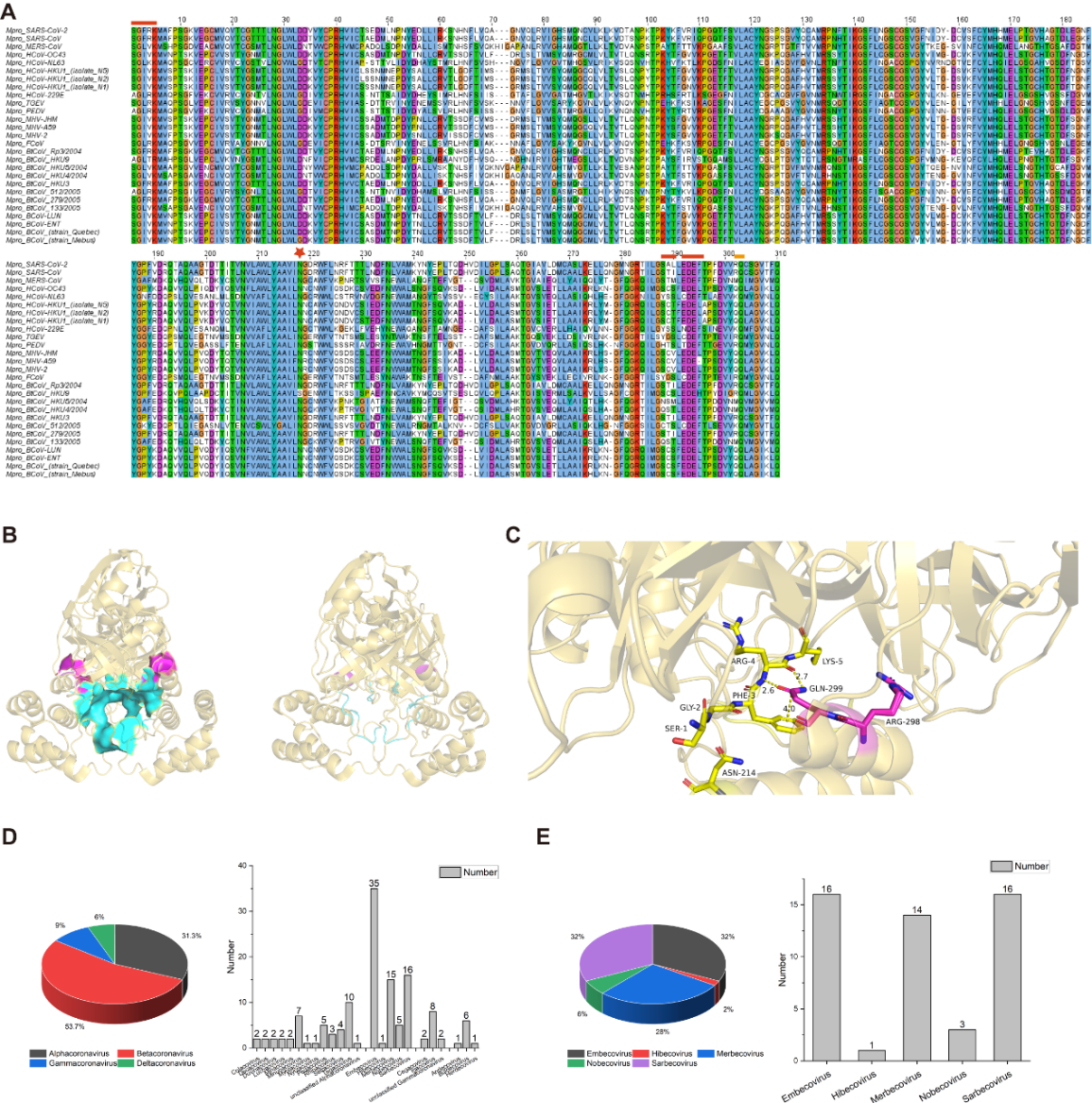


**Fig.** **S10** The conservation of Mpro and PLpro in coronaviruses. **A** The results of multiple sequence alignment against 27 Mpro sequences from the UniProt database, covering diverse coronaviruses. **B** The nano-channel of Mpro is displayed in both surface and cartoon representations. The N-terminal five residues (SGFRK), Asn214, and the region Glu288-Asp289-Glu290-Phe291 from the flank, as well as Ser284-Ala285-Leu286 from the bottom, are colored cyan. Arg298–Gln299 in the C-terminus residing around the nano-channel are colored magenta. The top of the nano-channel is open and connected with the inner cavities of Mpro. **C** The detailed interaction between Gln299 and the N-terminal residues. **B-C** The structure of SARS-CoV-2 Mpro is illustrated using the PDB model 6WTM. **D** Results of SARS-CoV-2 Mpro BLAST search in the UniProt database (identity ≥ 30% and E-value ≤ e^-5^). **E** Results of SARS-CoV-2 PLpro BLAST search in the UniProt database (identity ≥ 30% and E-value ≤ e^-5^).
